# Supplementary material for: Tunable Aryl Imidazolium Recyclable Ionic Liquid with Dual Brønsted–Lewis Acid as Green Catalyst for Friedel–Crafts Acylation and Thioesterification
Source: Molecules. 2020 Jan 15;25(2):352. doi: 10.3390/molecules25020352 (PMC7024267; doi:10.3390/molecules25020352)

Supporting Information

Tunable aryl imidazolium recyclable ionic liquid with dual Brønsted and Lewis acid as green catalyst for Friedel-Crafts acylation and thioesterification

Yi-Jyun Lin,[1] Yao-Peng Wu,[1] Mayur Thul,[1] Ming-Wei Hung,[1] Shih-Huan Chou,[2] Wen-Tin Chen,[3] Wesley Lin,[4] Michelle Lin,[4] Dagulla mallikarjuna reddy,[1] Hsin-Ru Wu,[5] Wen-Yueh Ho,*[,6] Shun-Yuan Luo*[,1]

[1]Department of Chemistry, National Chung Hsing University, Taichung 402, Taiwan

[2]Taipei American School, Taipei 111, Taiwan

[3]Taichung Municipal Chungming Senior High School, Taichung 403, Taiwan

[4]Morrison Academy, Taichung 406, Taiwan

[5]Instrumentation center of MOST, National Tsing Hua University, Hsinchu, 300, Taiwan

[6]Department of Cosmetic Science and Institute of Cosmetic Science, Chia Nan University of Pharmacy and Science, Tainan 717, Taiwan

E-mail: *sylup@dragon.nchu.edu.tw*

**Contents**	**Page**
NMR spectrum of important compounds	2-43

	Parameter	Value
1	Origin	Varian
2	Spectrometer	vnmrs
3	Solvent	cdcl3
4	Temperature	20.0
5	Pulse Sequence	s2pul
6	Experiment	1D
7	Number of Scans	96
8	Receiver Gain	18
9	Relaxation Delay	1.0000
10	Acquisition Time	2.5559
11	Spectrometer Frequency	399.76
12	Spectral Width	6410.3
13	Lowest Frequency	-798.6
14	Nucleus	1H

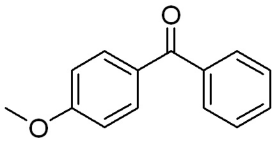

4a

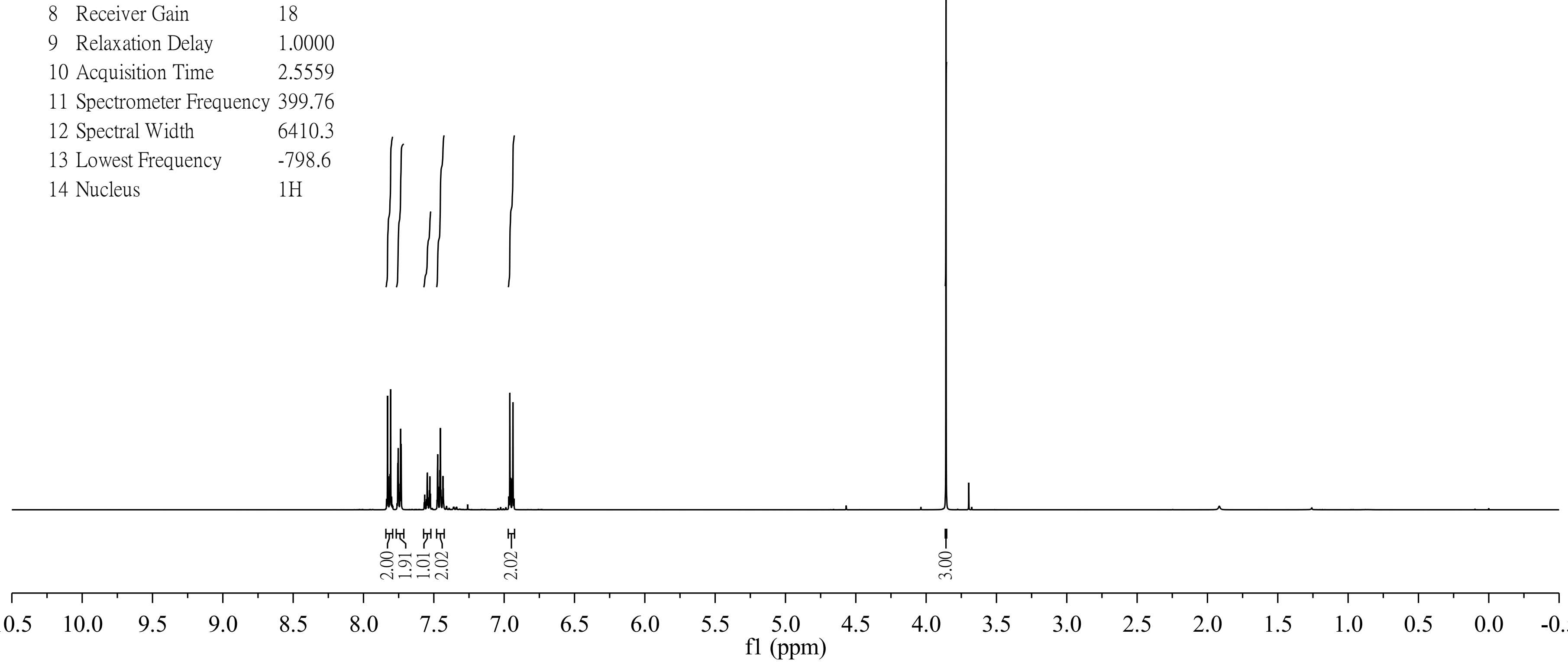

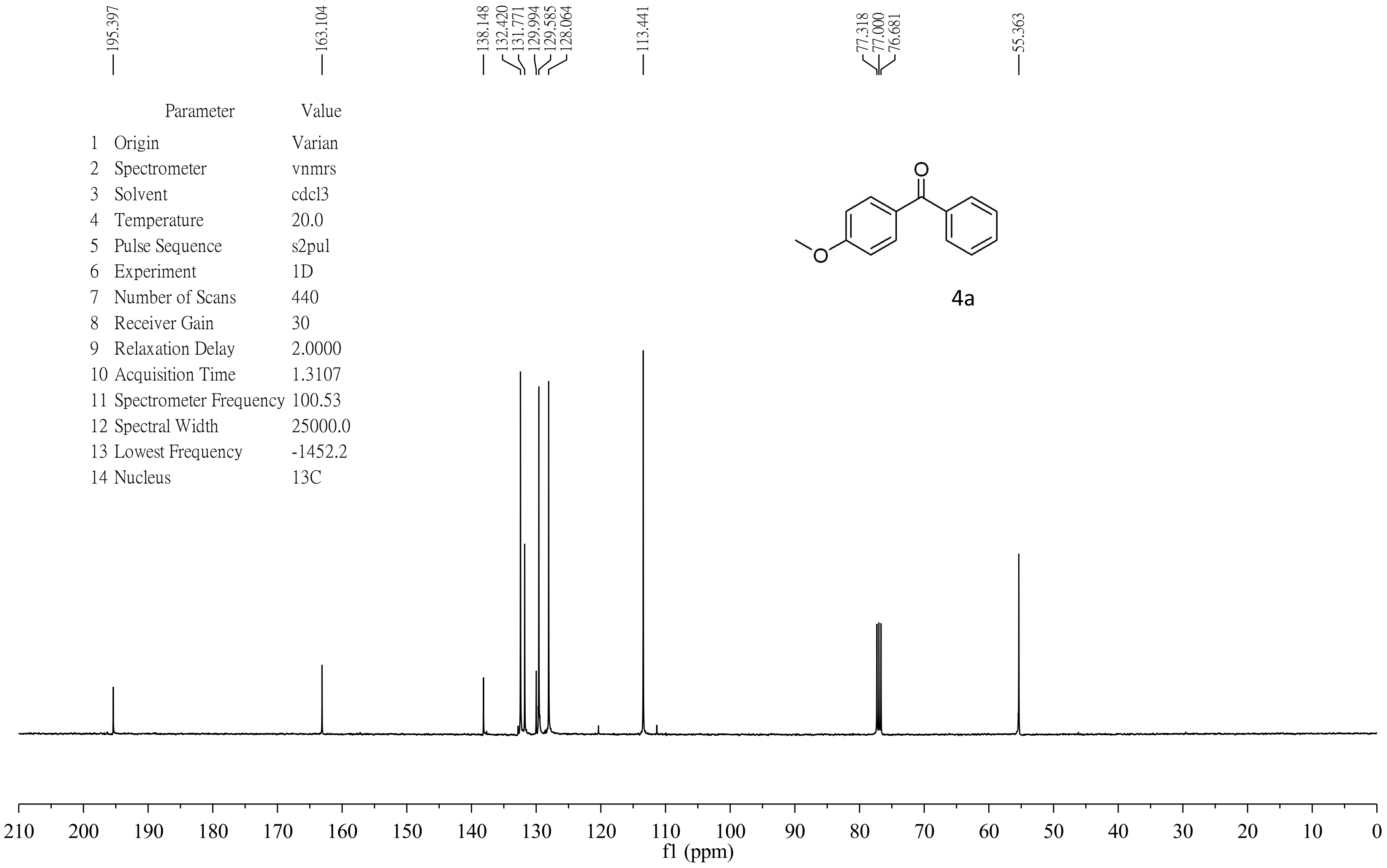
195.397
163.104
138.148
132.420
131.771
129.994
129.585
128.064
113.441
77.318
77.000
76.681
55.363
Parameter Value
1 Origin Varian
2 Spectrometer vnmrs
3 Solvent cdcl3
4 Temperature 20.0
5 Pulse Sequence s2pul
6 Experiment 1D
7 Number of Scans 440
8 Receiver Gain 30
9 Relaxation Delay 2.0000
10 Acquisition Time 1.3107
11 Spectrometer Frequency 100.53
12 Spectral Width 25000.0
13 Lowest Frequency -1452.2
14 Nucleus 13C
4a
210 200 190 180 170 160 150 140 130 120 110 100 90 80 70 60 50 40 30 20 10 0
f1 (ppm)

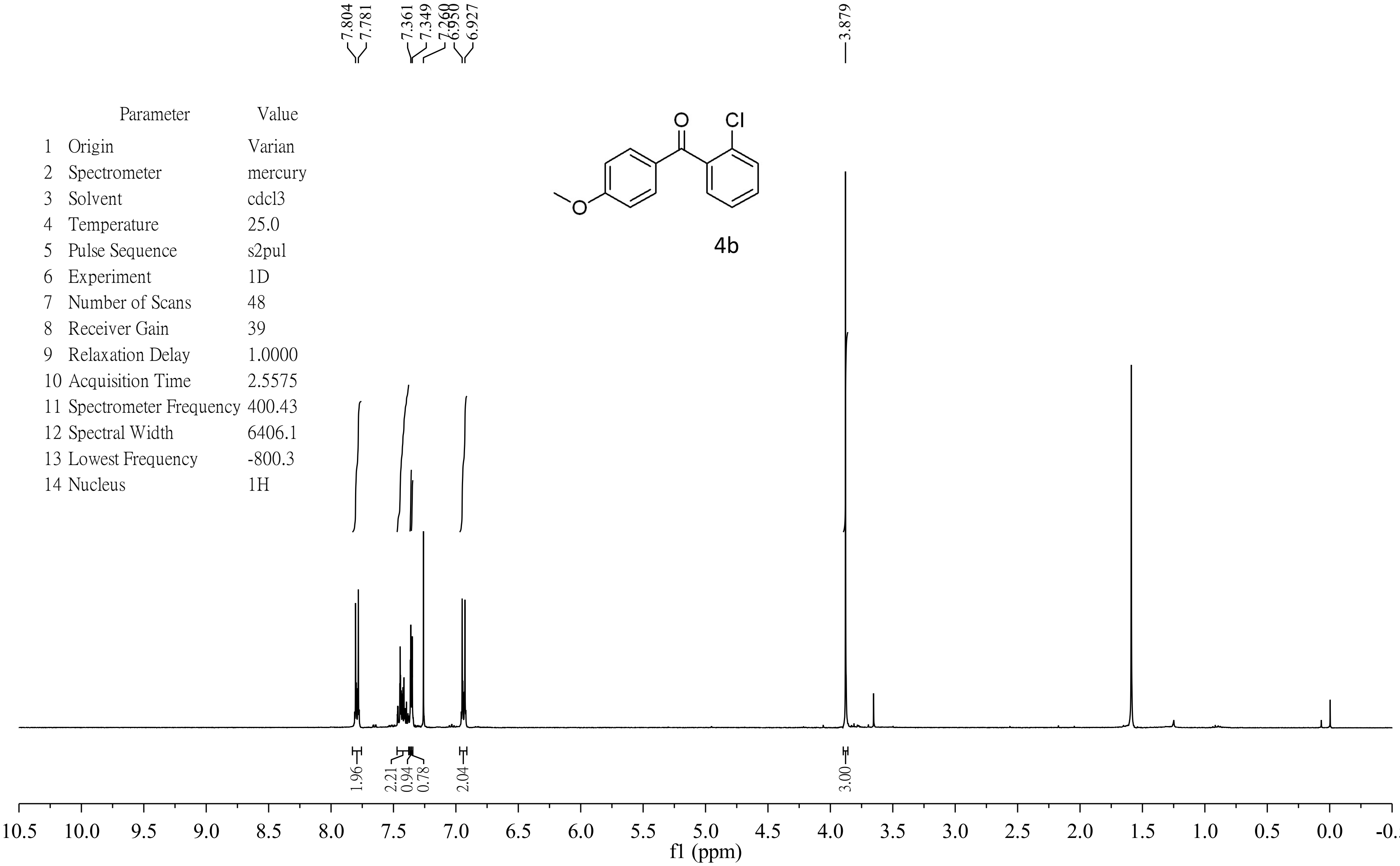
7.804
7.781
7.361
7.349
7.260
6.950
6.927
3.879
Parameter Value
1 Origin Varian
2 Spectrometer mercury
3 Solvent cdcl3
4 Temperature 25.0
5 Pulse Sequence s2pul
6 Experiment 1D
7 Number of Scans 48
8 Receiver Gain 39
9 Relaxation Delay 1.0000
10 Acquisition Time 2.5575
11 Spectrometer Frequency 400.43
12 Spectral Width 6406.1
13 Lowest Frequency -800.3
14 Nucleus 1H
O
Cl
4b
1.96
2.21
0.94
0.78
2.04
3.00
10.5 10.0 9.5 9.0 8.5 8.0 7.5 7.0 6.5 6.0 5.5 5.0 4.5 4.0 3.5 3.0 2.5 2.0 1.5 1.0 0.5 0.0 -0.5
f1 (ppm)

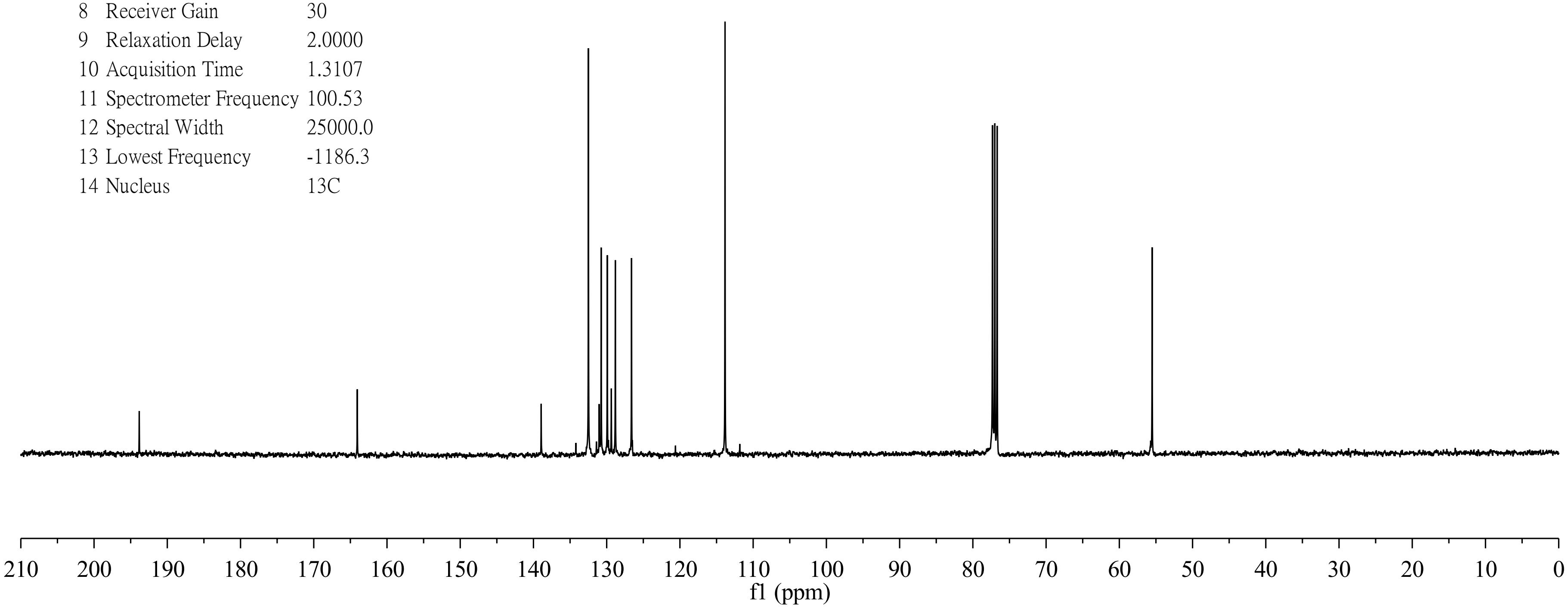

	Parameter	Value
1	Origin	Varian
2	Spectrometer	vnmrs
3	Solvent	d2o
4	Temperature	20.0
5	Pulse Sequence	s2pul
6	Experiment	1D
7	Number of Scans	888
8	Receiver Gain	30
9	Relaxation Delay	2.0000
10	Acquisition Time	1.3107
11	Spectrometer Frequency	100.53
12	Spectral Width	25000.0
13	Lowest Frequency	-1186.3
14	Nucleus	13C

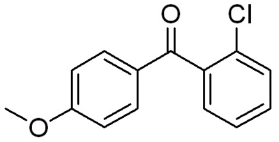

4b

	Parameter	Value
1	Origin	Varian
2	Spectrometer	mercury
3	Solvent	cdcl3
4	Temperature	25.0
5	Pulse Sequence	s2pul
6	Experiment	1D
7	Number of Scans	120
8	Receiver Gain	39
9	Relaxation Delay	1.0000
10	Acquisition Time	2.5575
11	Spectrometer Frequency	400.43
12	Spectral Width	6406.1
13	Lowest Frequency	-800.0
14	Nucleus	1H

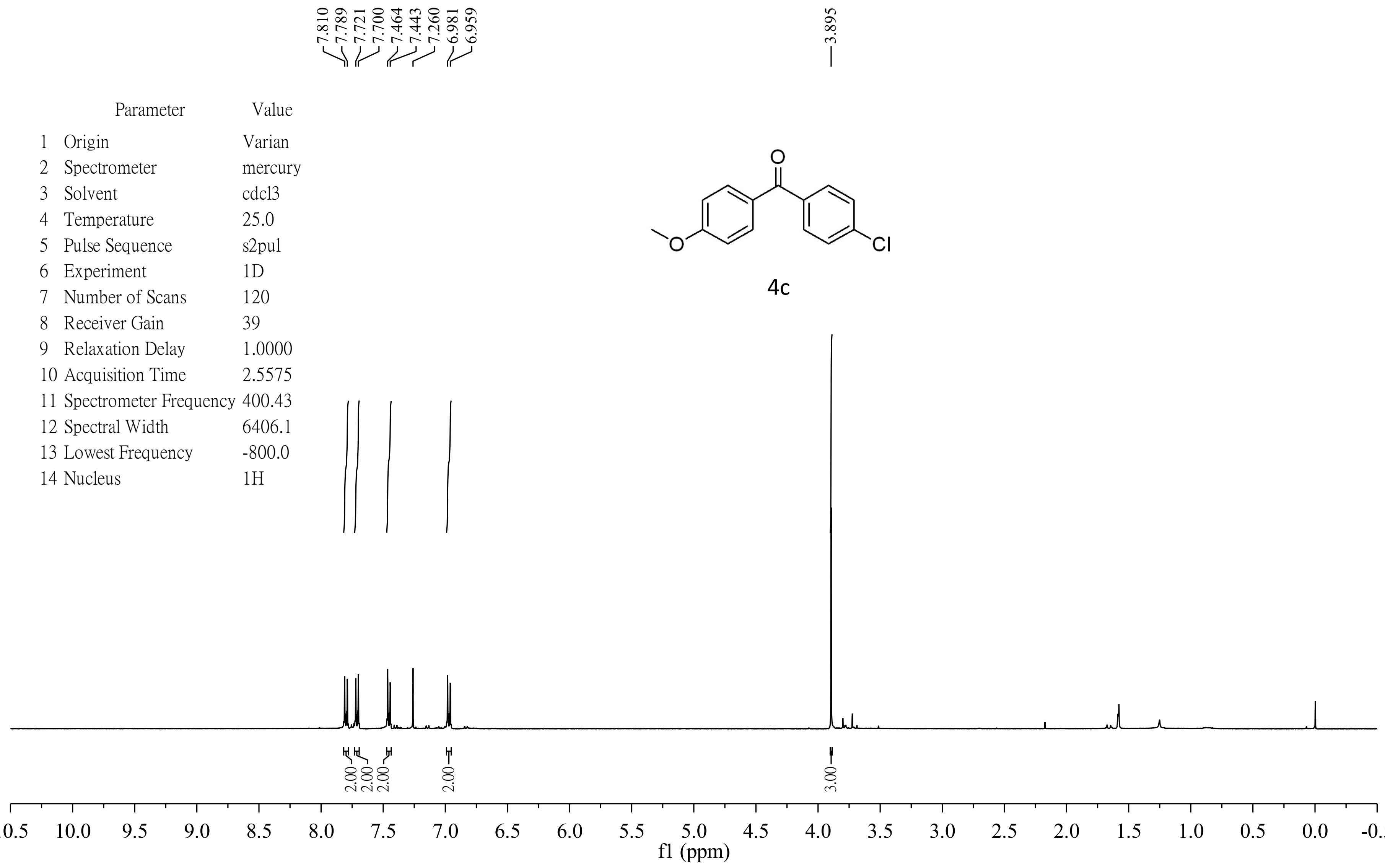

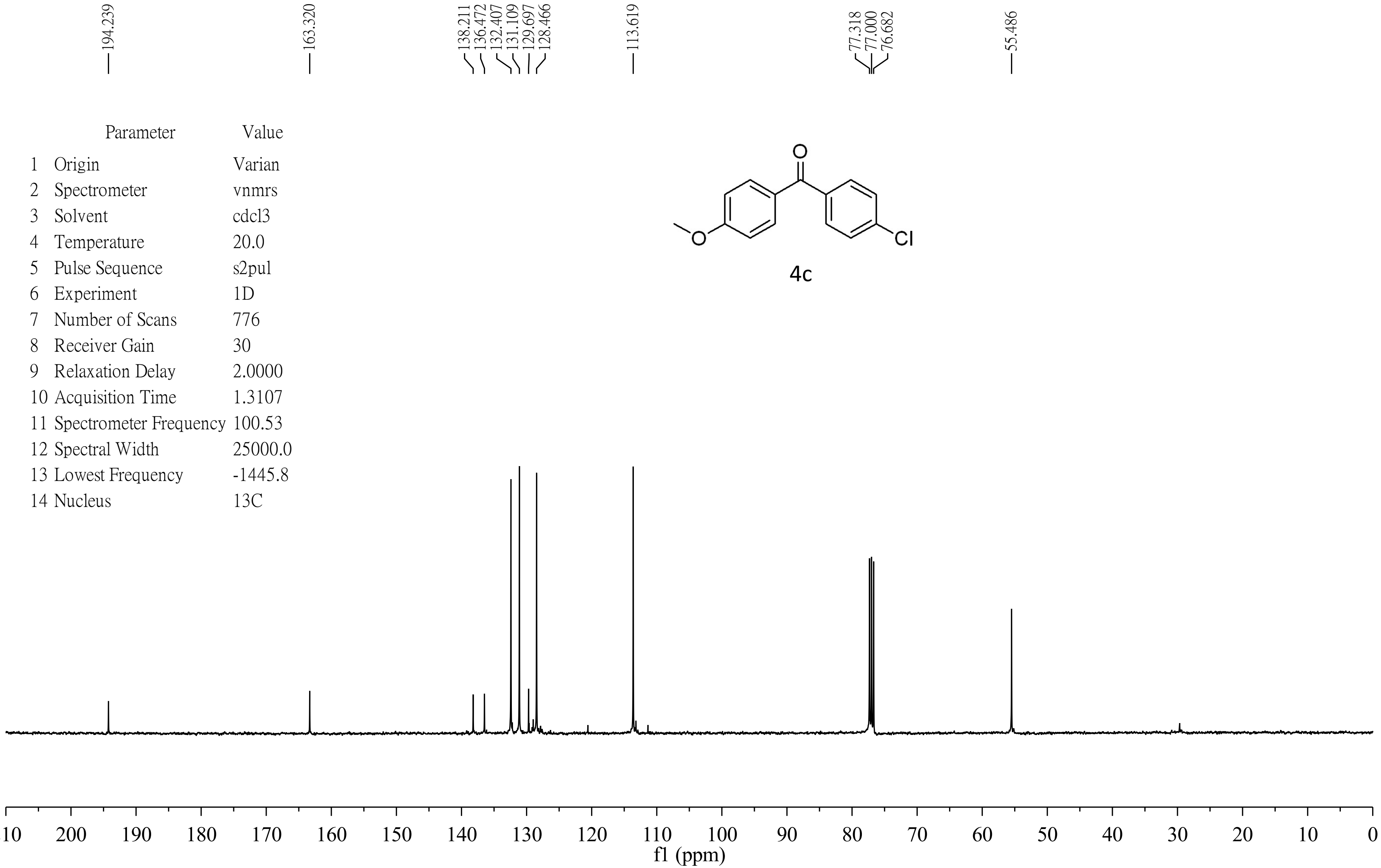

	Parameter	Value
1	Origin	Varian
2	Spectrometer	vnmrs
3	Solvent	cdcl3
4	Temperature	20.0
5	Pulse Sequence	s2pul
6	Experiment	1D
7	Number of Scans	776
8	Receiver Gain	30
9	Relaxation Delay	2.0000
10	Acquisition Time	1.3107
11	Spectrometer Frequency	100.53
12	Spectral Width	25000.0
13	Lowest Frequency	-1445.8
14	Nucleus	13C

4c

	Parameter	Value
1	Origin	Varian
2	Spectrometer	vnmrs
3	Solvent	cdcl3
4	Temperature	20.0
5	Pulse Sequence	s2pul
6	Experiment	1D
7	Number of Scans	44
8	Receiver Gain	30
9	Relaxation Delay	1.0000
10	Acquisition Time	2.5559
11	Spectrometer Frequency	399.76
12	Spectral Width	6410.3
13	Lowest Frequency	-806.6
14	Nucleus	1H

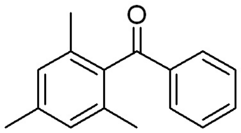

4d

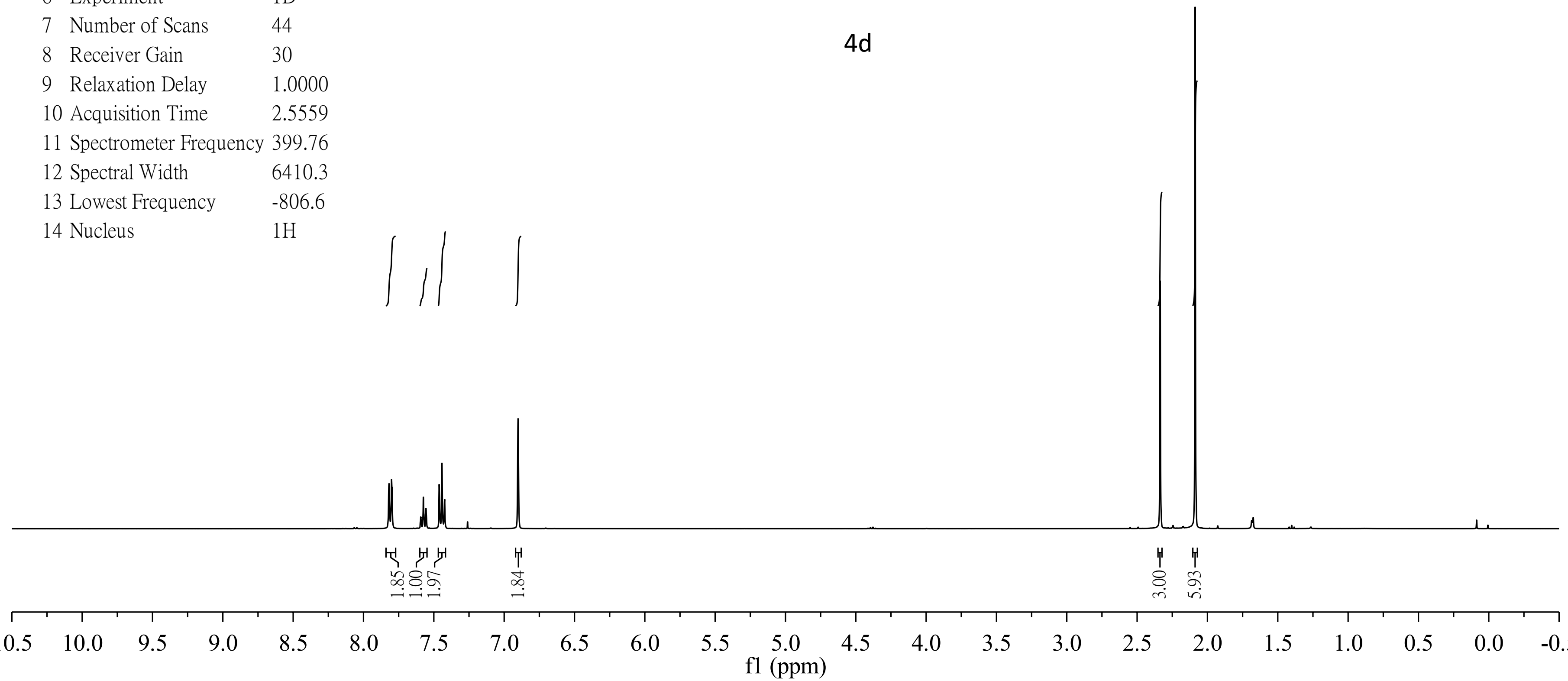

	Parameter	Value
1	Origin	Varian
2	Spectrometer	vnmrs
3	Solvent	cdcl3
4	Temperature	20.0
5	Pulse Sequence	s2pul
6	Experiment	1D
7	Number of Scans	188
8	Receiver Gain	30
9	Relaxation Delay	2.0000
10	Acquisition Time	1.3107
11	Spectrometer Frequency	100.53
12	Spectral Width	25000.0
13	Lowest Frequency	-1443.1
14	Nucleus	13C

4d

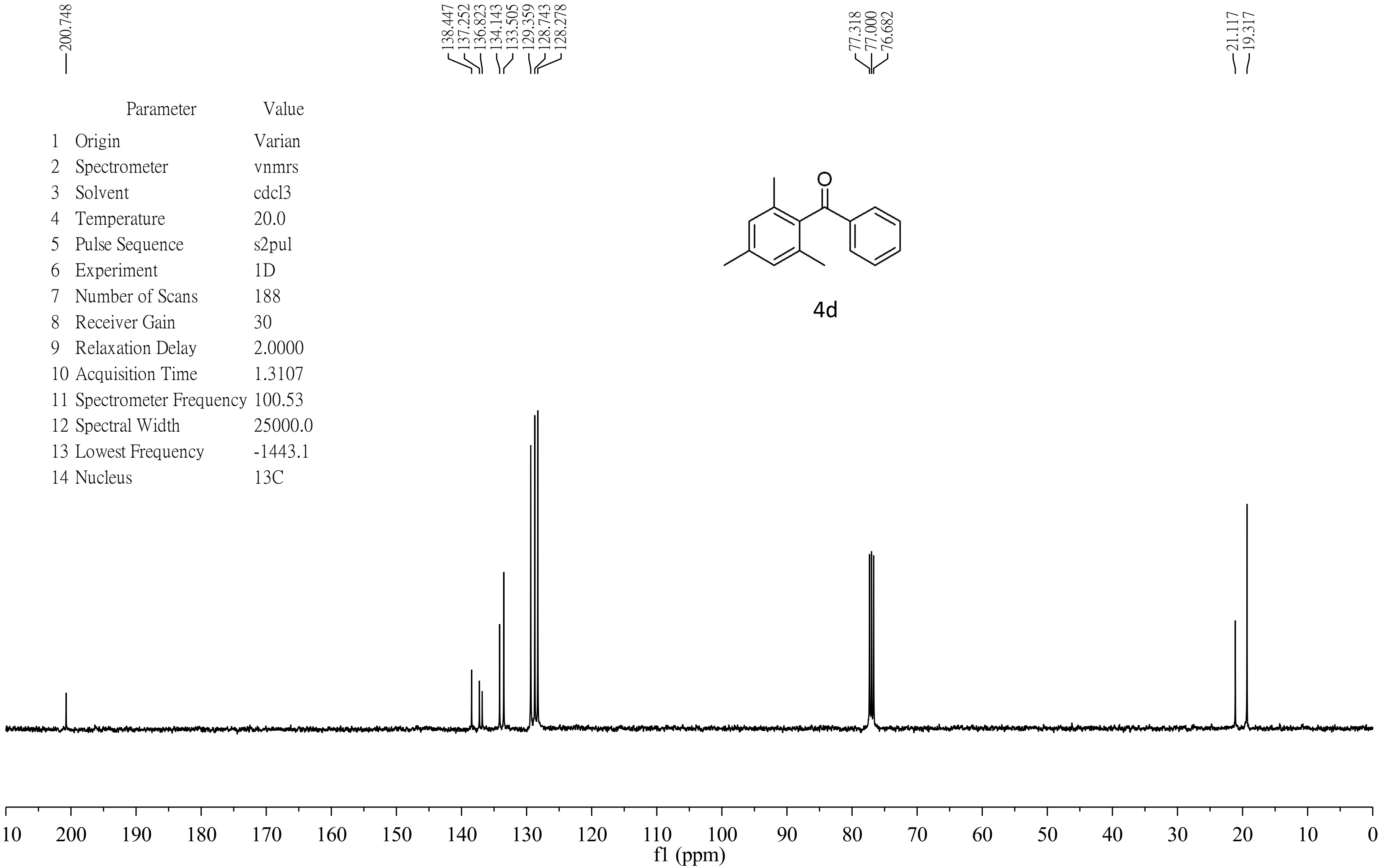

	Parameter	Value
1	Origin	Varian
2	Spectrometer	vnmrs
3	Solvent	cdcl3
4	Temperature	20.0
5	Pulse Sequence	s2pul
6	Experiment	1D
7	Number of Scans	36
8	Receiver Gain	30
9	Relaxation Delay	1.0000
10	Acquisition Time	2.5559
11	Spectrometer Frequency	399.76
12	Spectral Width	6410.3
13	Lowest Frequency	-806.6
14	Nucleus	1H

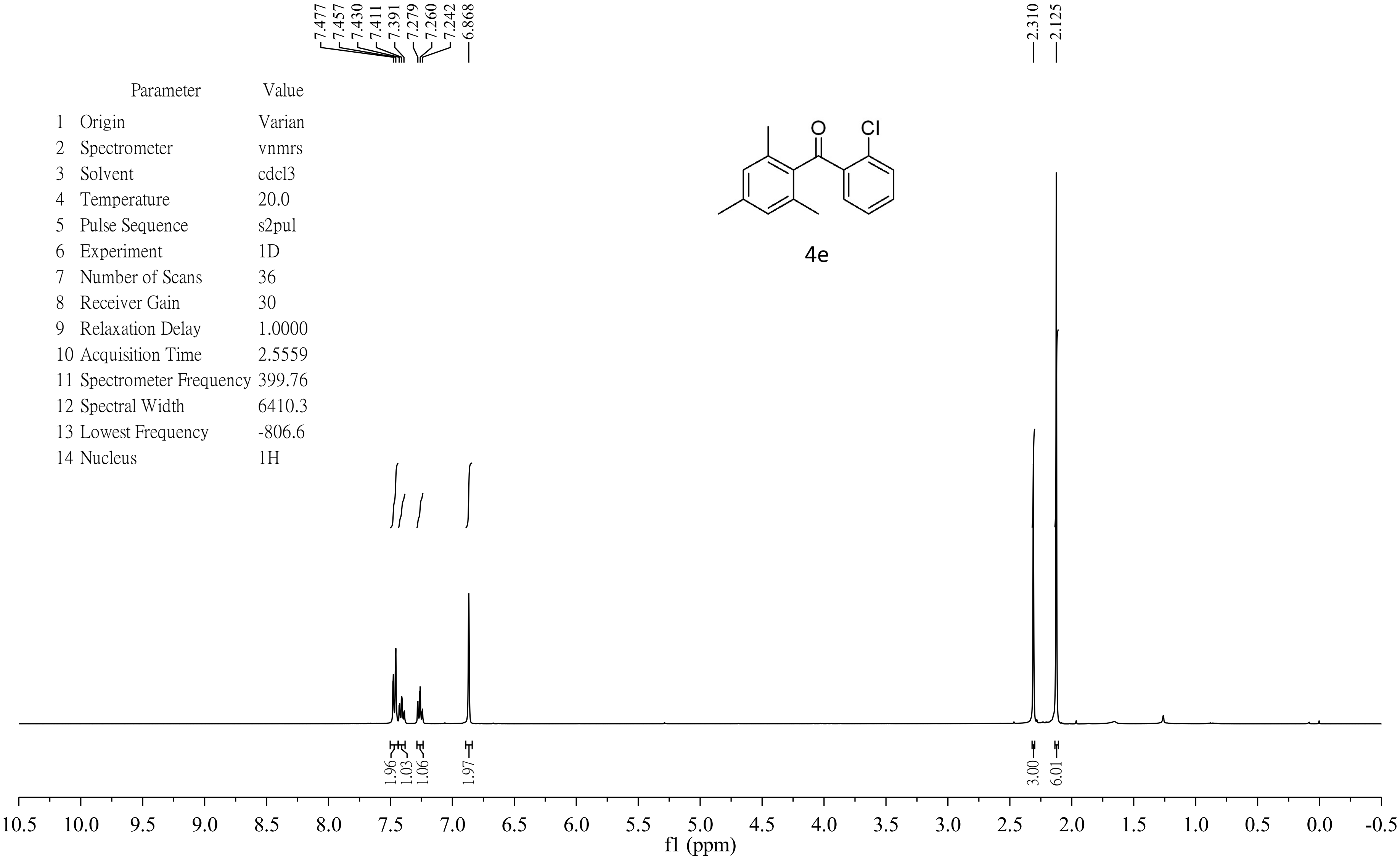

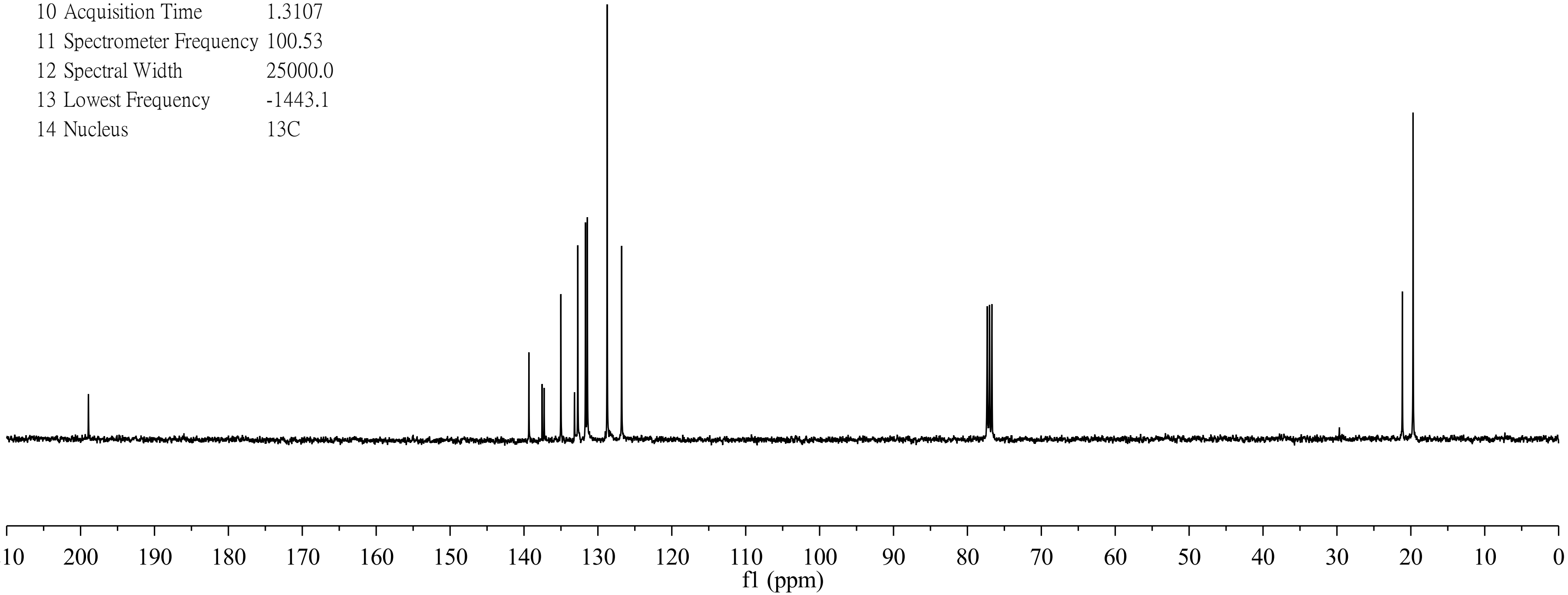

	Parameter	Value
1	Origin	Varian
2	Spectrometer	vnmrs
3	Solvent	cdcl3
4	Temperature	20.0
5	Pulse Sequence	s2pul
6	Experiment	1D
7	Number of Scans	116
8	Receiver Gain	30
9	Relaxation Delay	2.0000
10	Acquisition Time	1.3107
11	Spectrometer Frequency	100.53
12	Spectral Width	25000.0
13	Lowest Frequency	-1443.1
14	Nucleus	13C

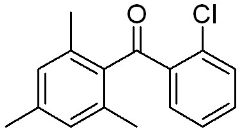

4e

	Parameter	Value
1	Origin	Varian
2	Spectrometer	vnmrs
3	Solvent	cdcl3
4	Temperature	20.0
5	Pulse Sequence	s2pul
6	Experiment	1D
7	Number of Scans	36
8	Receiver Gain	30
9	Relaxation Delay	1.0000
10	Acquisition Time	2.5559
11	Spectrometer Frequency	399.76
12	Spectral Width	6410.3
13	Lowest Frequency	-798.6
14	Nucleus	1H

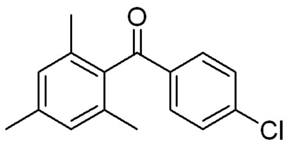

4f

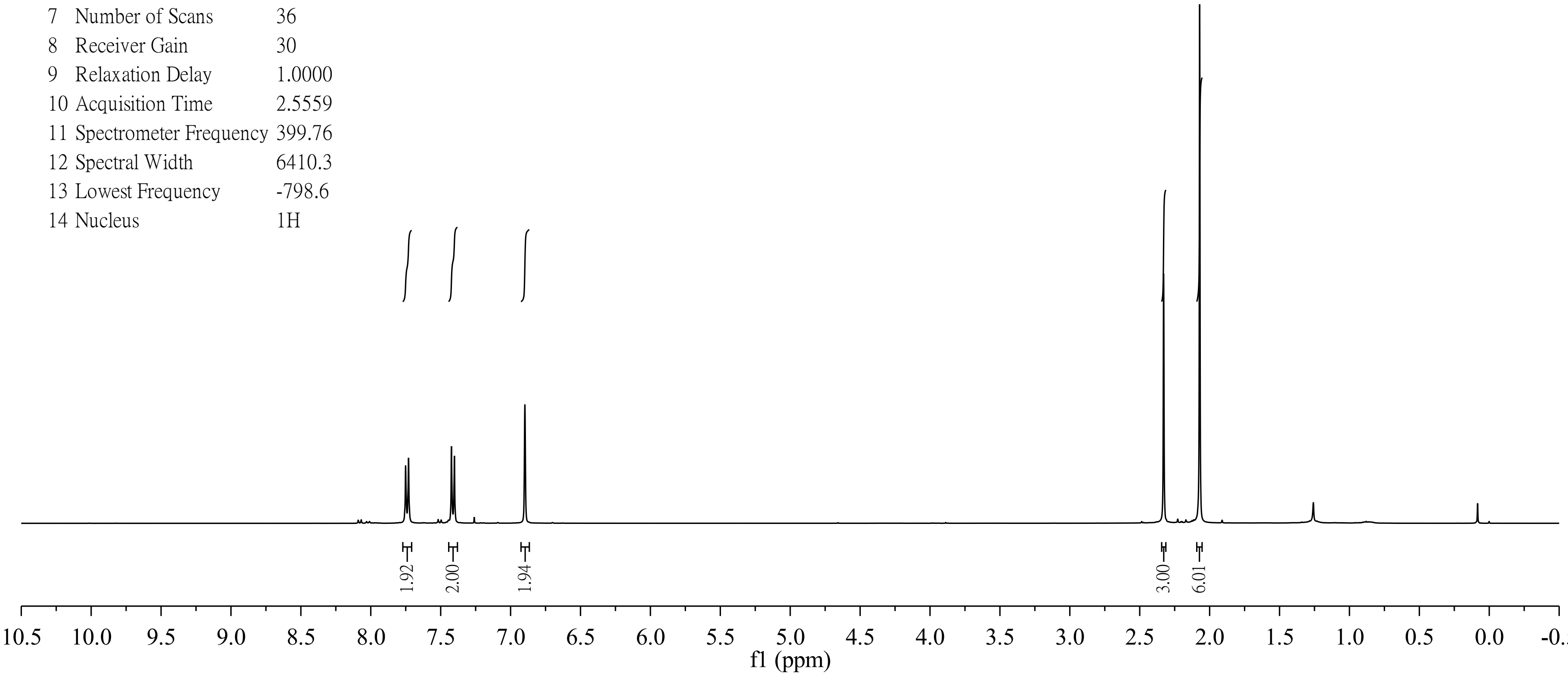

	Parameter	Value
1	Origin	Varian
2	Spectrometer	vnmrs
3	Solvent	cdcl3
4	Temperature	20.0
5	Pulse Sequence	s2pul
6	Experiment	1D
7	Number of Scans	164
8	Receiver Gain	30
9	Relaxation Delay	2.0000
10	Acquisition Time	1.3107
11	Spectrometer Frequency	100.53
12	Spectral Width	25000.0
13	Lowest Frequency	-1444.7
14	Nucleus	13C

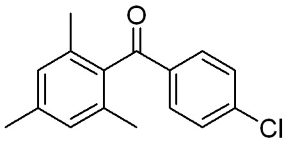

4f

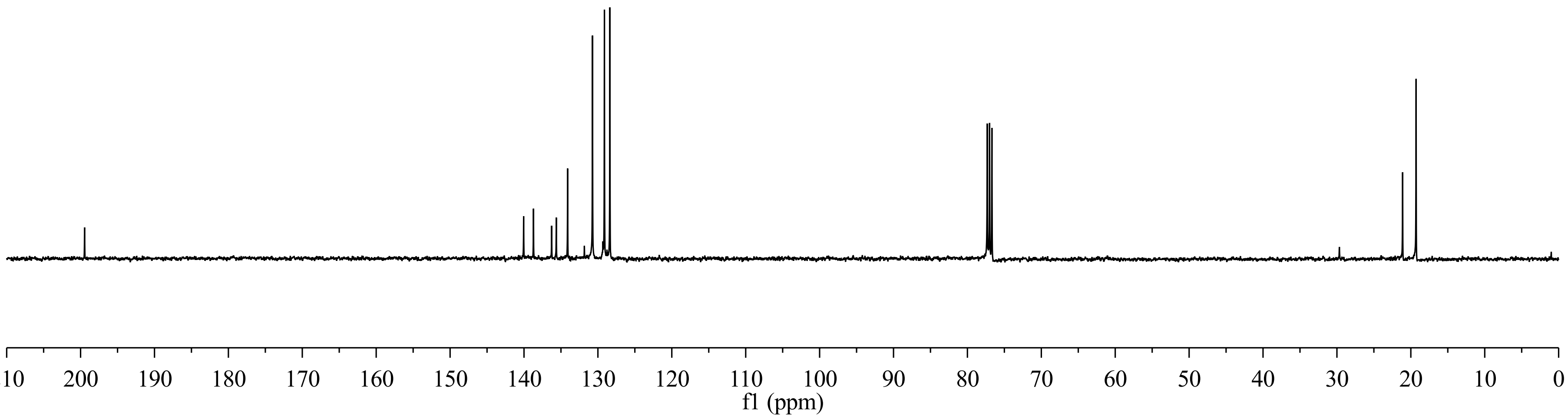

	Parameter	Value
1	Origin	Varian
2	Spectrometer	vnmrs
3	Solvent	cdcl3
4	Temperature	20.0
5	Pulse Sequence	s2pul
6	Experiment	1D
7	Number of Scans	80
8	Receiver Gain	2
9	Relaxation Delay	1.0000
10	Acquisition Time	2.5559
11	Spectrometer Frequency	399.76
12	Spectral Width	6410.3
13	Lowest Frequency	-798.6
14	Nucleus	1H

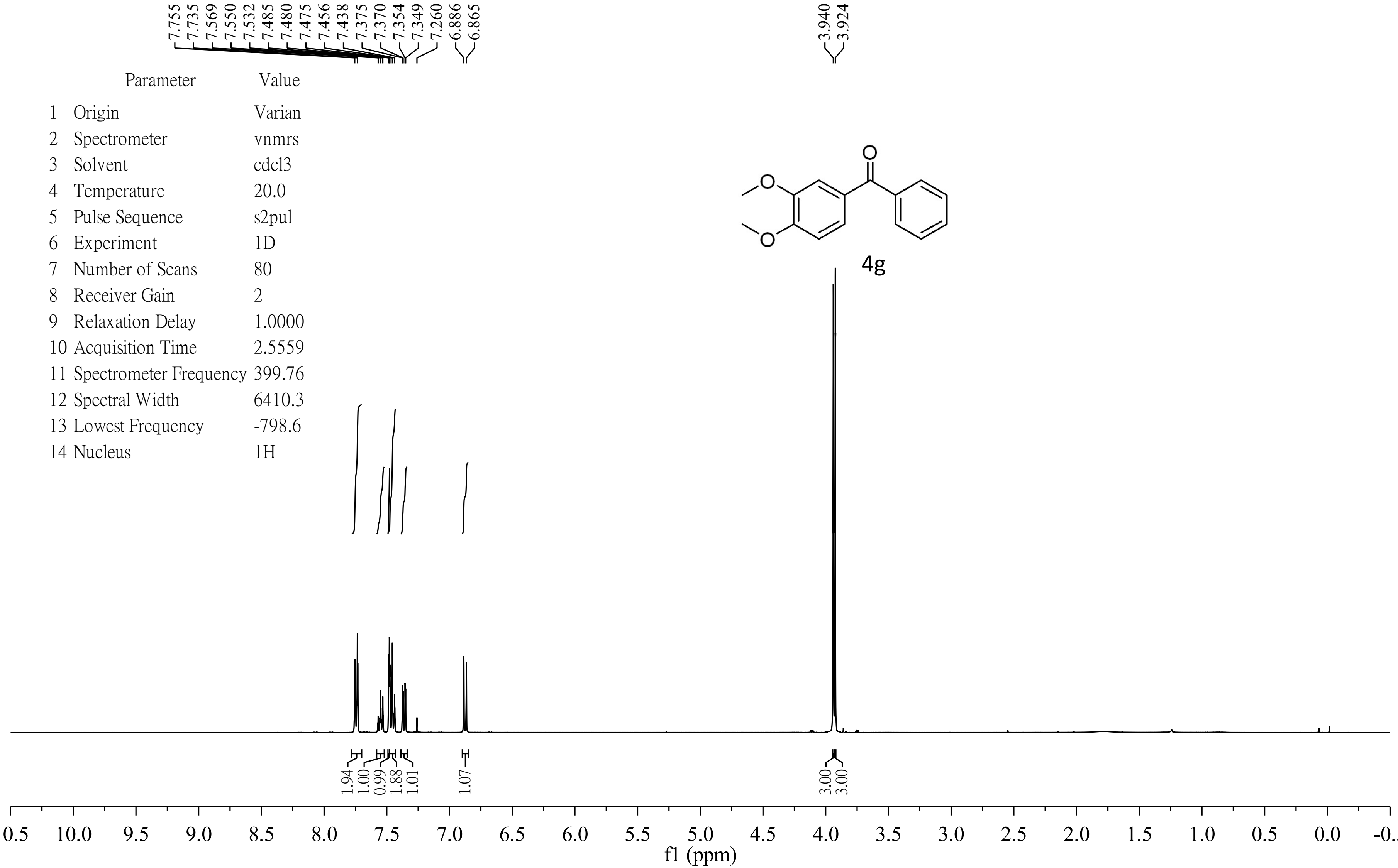

4g

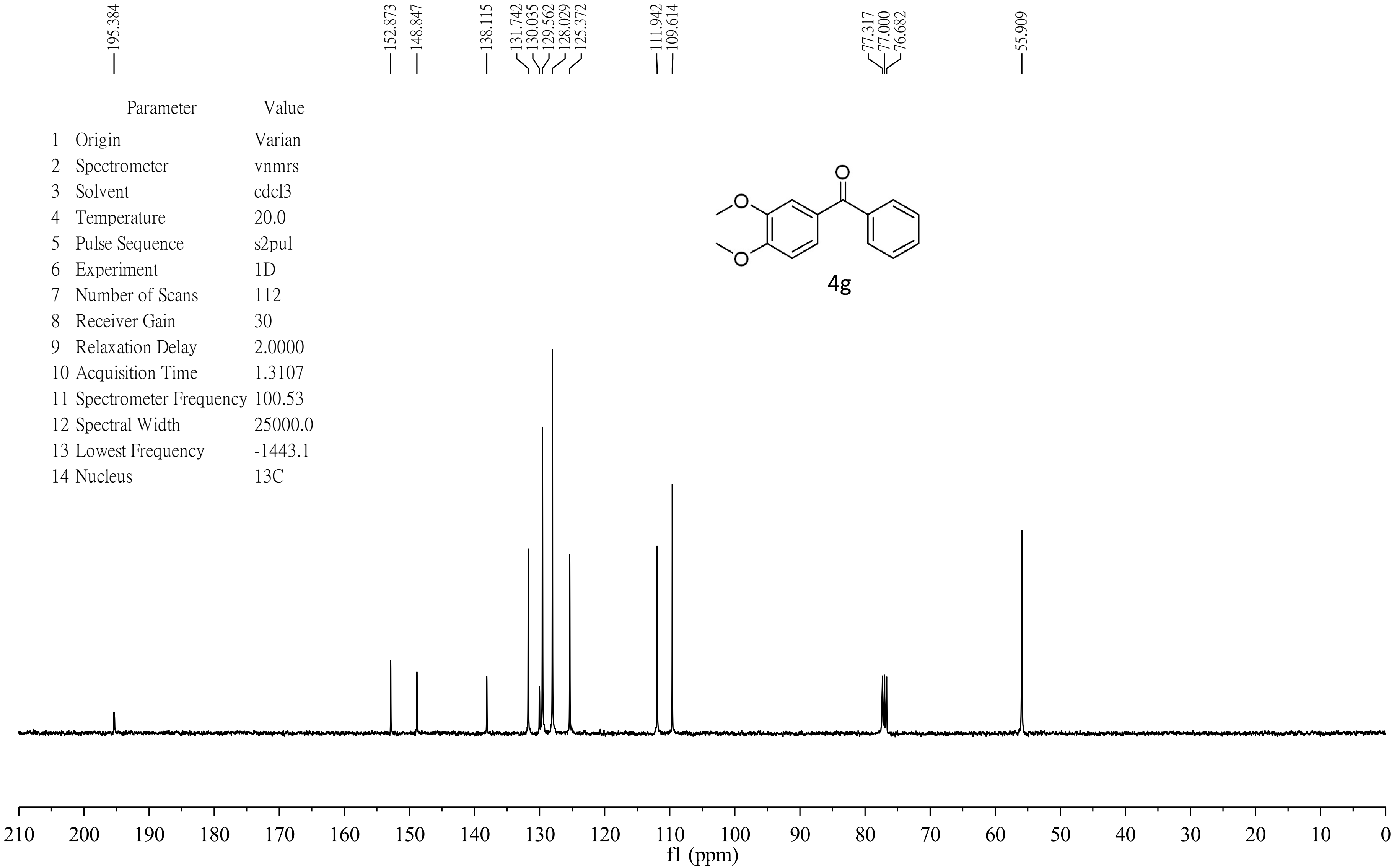
195.384
152.873
148.847
138.115
131.742
130.035
129.562
128.029
125.372
111.942
109.614
77.317
77.000
76.682
55.909
Parameter Value
1 Origin Varian
2 Spectrometer vnmrs
3 Solvent cdcl3
4 Temperature 20.0
5 Pulse Sequence s2pul
6 Experiment 1D
7 Number of Scans 112
8 Receiver Gain 30
9 Relaxation Delay 2.0000
10 Acquisition Time 1.3107
11 Spectrometer Frequency 100.53
12 Spectral Width 25000.0
13 Lowest Frequency -1443.1
14 Nucleus 13C
O
O
O
4g
210 200 190 180 170 160 150 140 130 120 110 100 90 80 70 60 50 40 30 20 10 0
f1 (ppm)

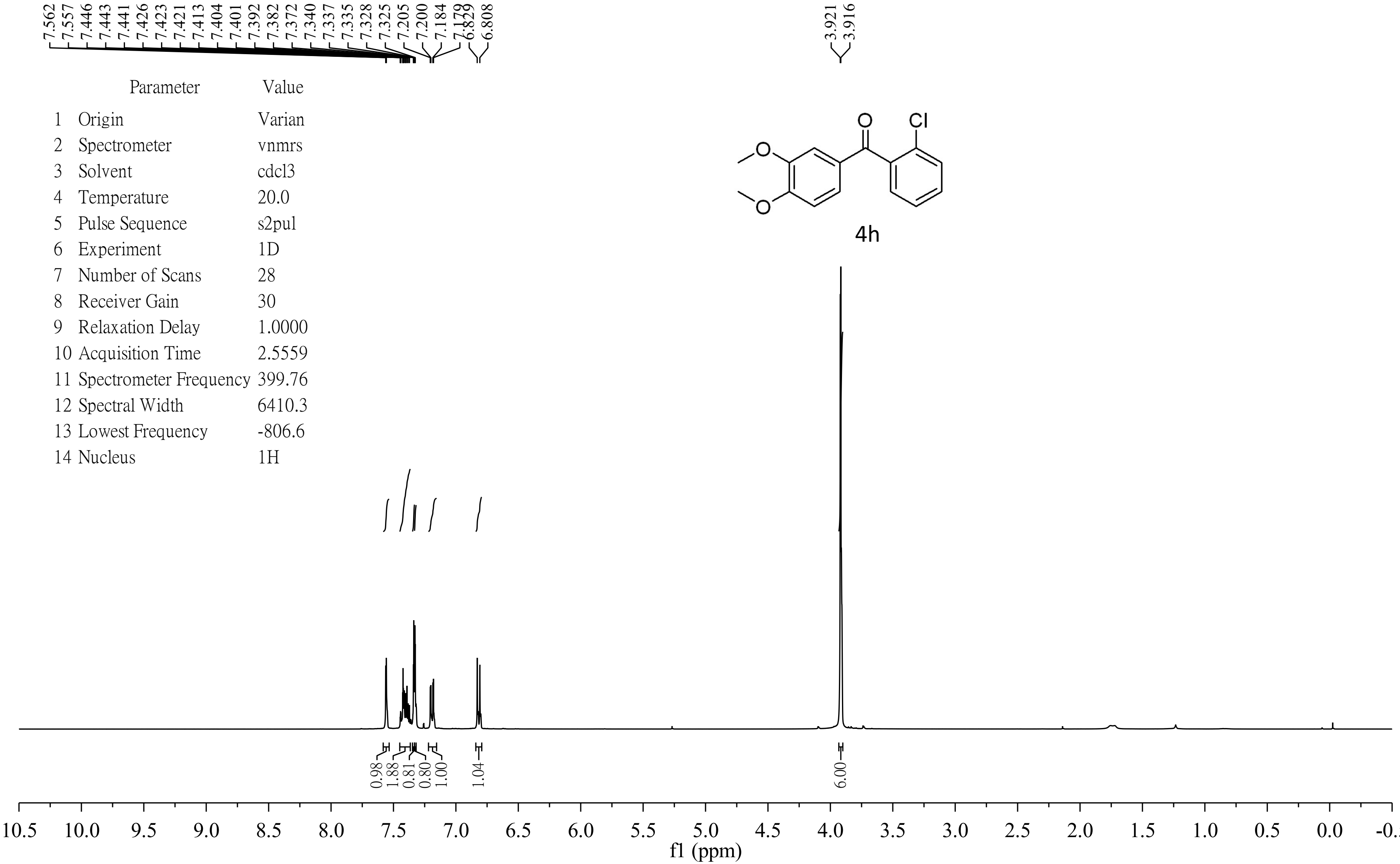

	Parameter	Value
1	Origin	Varian
2	Spectrometer	vnmrs
3	Solvent	cdcl3
4	Temperature	20.0
5	Pulse Sequence	s2pul
6	Experiment	1D
7	Number of Scans	28
8	Receiver Gain	30
9	Relaxation Delay	1.0000
10	Acquisition Time	2.5559
11	Spectrometer Frequency	399.76
12	Spectral Width	6410.3
13	Lowest Frequency	-806.6
14	Nucleus	1H

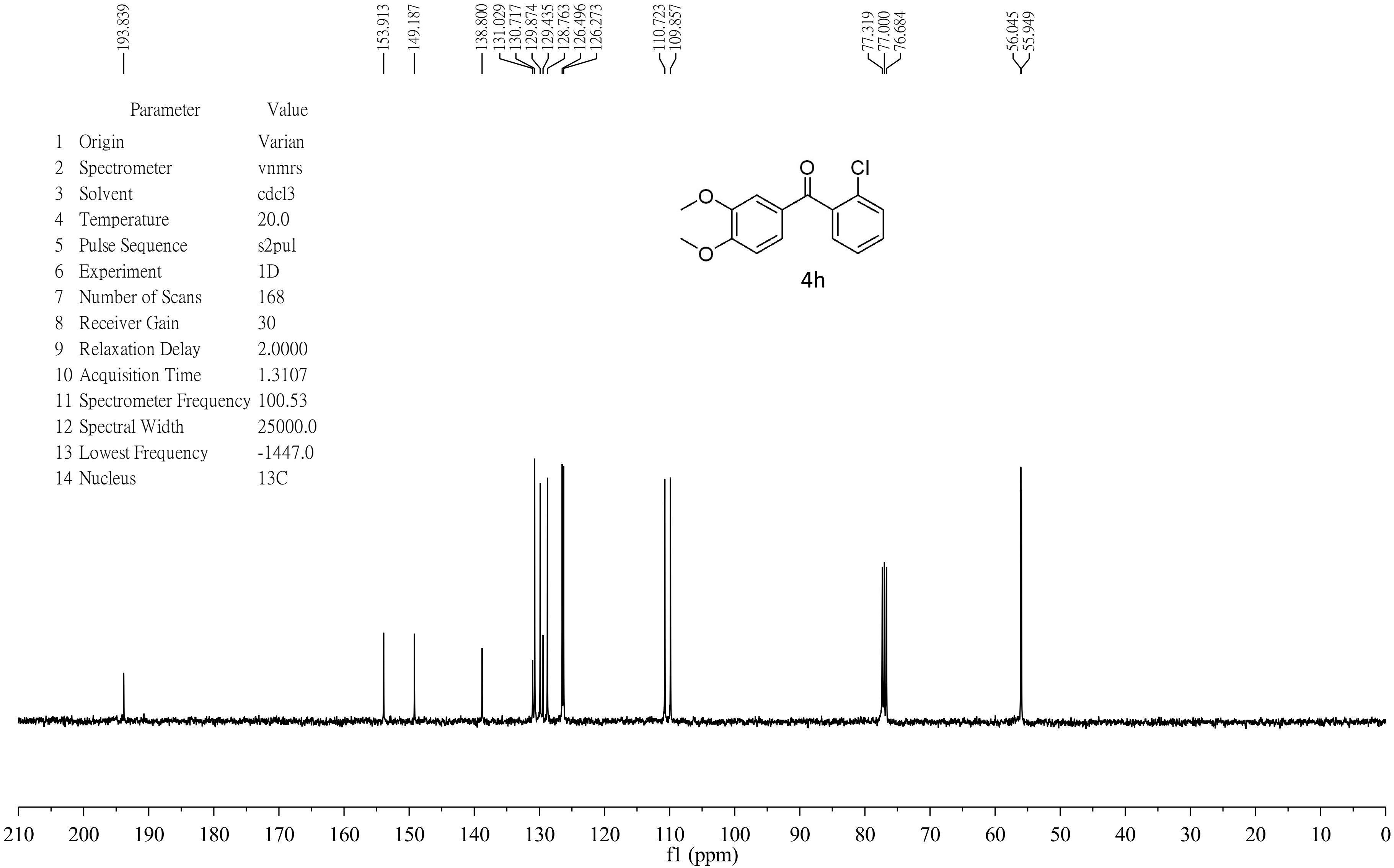

	Parameter	Value
1	Origin	Varian
2	Spectrometer	vnmrs
3	Solvent	cdcl3
4	Temperature	20.0
5	Pulse Sequence	s2pul
6	Experiment	1D
7	Number of Scans	168
8	Receiver Gain	30
9	Relaxation Delay	2.0000
10	Acquisition Time	1.3107
11	Spectrometer Frequency	100.53
12	Spectral Width	25000.0
13	Lowest Frequency	-1447.0
14	Nucleus	13C

4h

	Parameter	Value
1	Origin	Varian
2	Spectrometer	vnmrs
3	Solvent	cdcl3
4	Temperature	20.0
5	Pulse Sequence	s2pul
6	Experiment	1D
7	Number of Scans	24
8	Receiver Gain	52
9	Relaxation Delay	1.0000
10	Spectrometer Frequency	399.76
11	Spectral Width	6410.3
12	Lowest Frequency	-806.6
13	Nucleus	1H

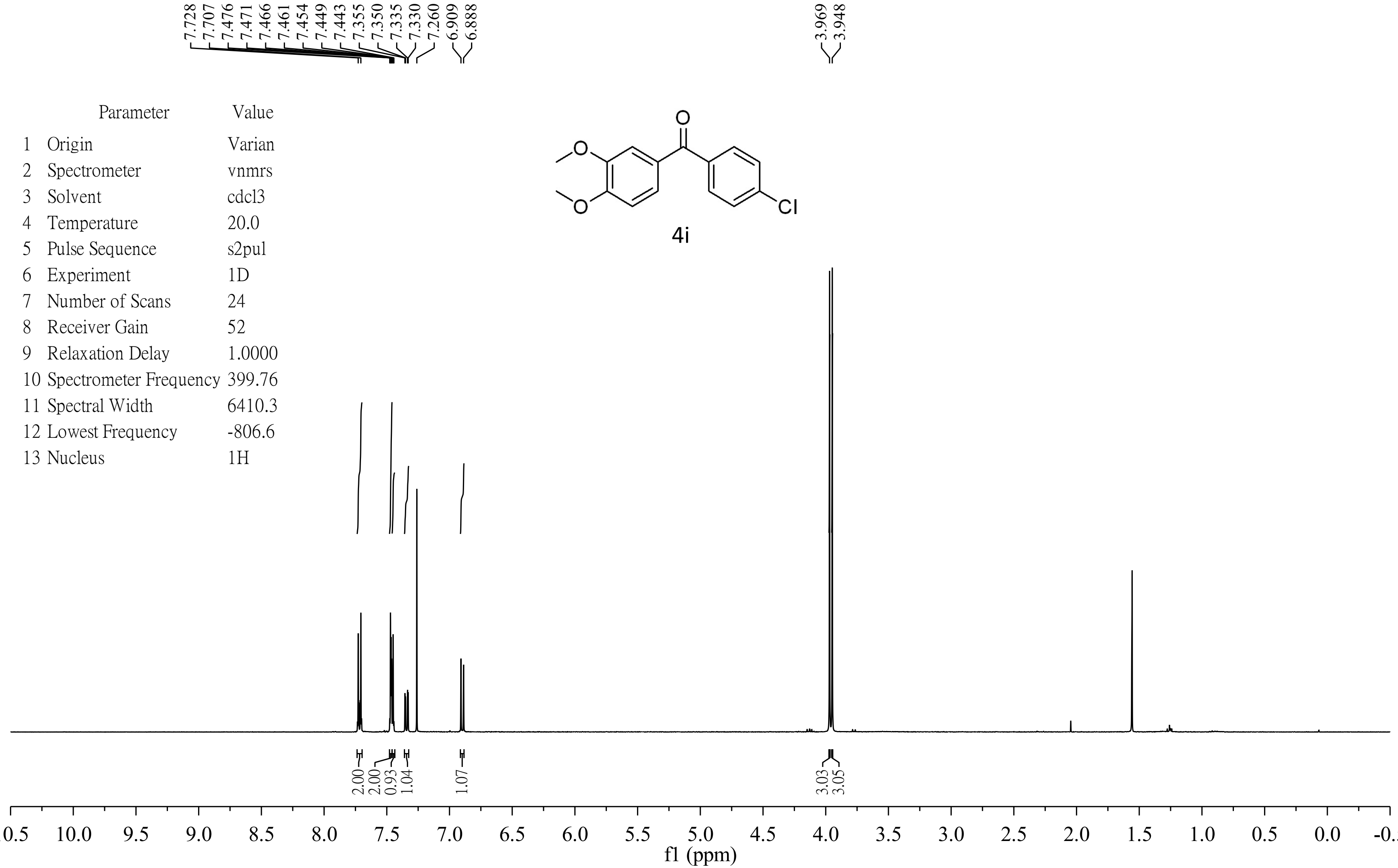

4i

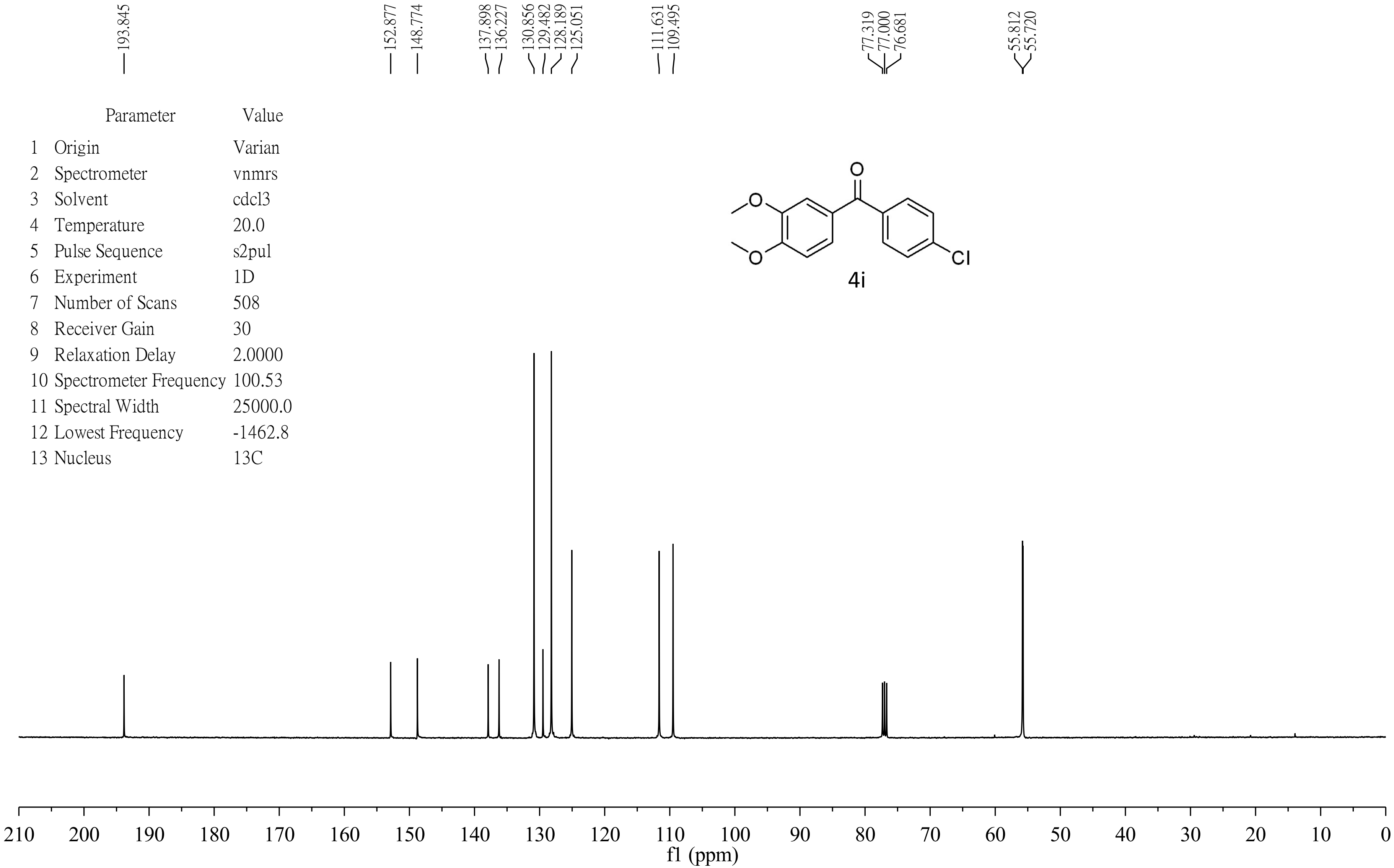

	Parameter	Value
1	Origin	Varian
2	Spectrometer	vnmrs
3	Solvent	cdcl3
4	Temperature	20.0
5	Pulse Sequence	s2pul
6	Experiment	1D
7	Number of Scans	508
8	Receiver Gain	30
9	Relaxation Delay	2.0000
10	Spectrometer Frequency	100.53
11	Spectral Width	25000.0
12	Lowest Frequency	-1462.8
13	Nucleus	13C

	Parameter	Value
1	Origin	Varian
2	Spectrometer	vnmrs
3	Solvent	cdcl3
4	Temperature	20.0
5	Pulse Sequence	s2pul
6	Experiment	1D
7	Number of Scans	56
8	Receiver Gain	18
9	Relaxation Delay	1.0000
10	Acquisition Time	2.5559
11	Spectrometer Frequency	399.76
12	Spectral Width	6410.3
13	Lowest Frequency	-799.0
14	Nucleus	1H

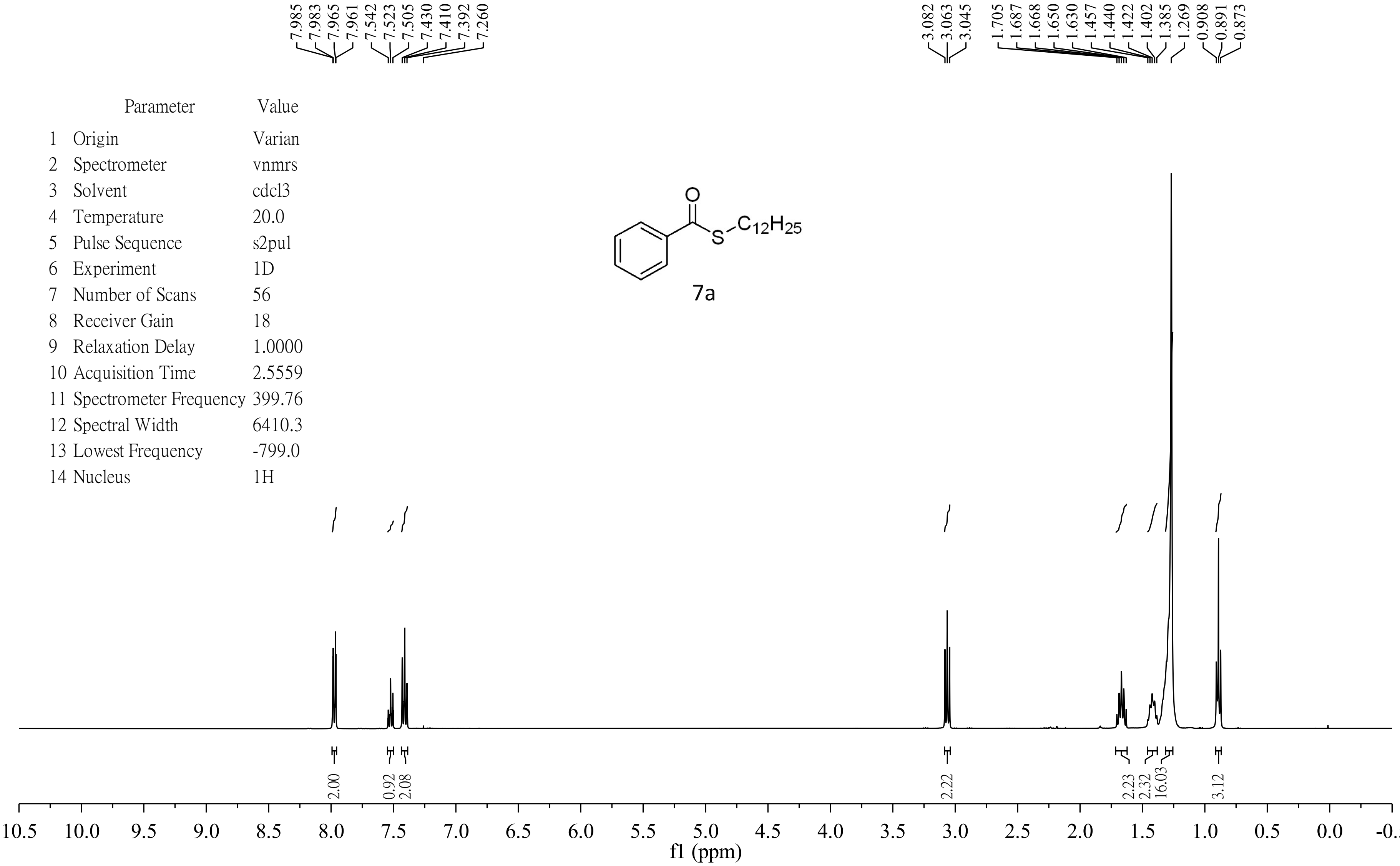

	Parameter	Value
1	Origin	Varian
2	Spectrometer	vnmrs
3	Solvent	cdcl3
4	Temperature	20.0
5	Pulse Sequence	s2pul
6	Experiment	1D
7	Number of Scans	1040
8	Receiver Gain	30
9	Relaxation Delay	2.0000
10	Acquisition Time	1.3107
11	Spectrometer Frequency	100.53
12	Spectral Width	25000.0
13	Lowest Frequency	-1453.4
14	Nucleus	13C

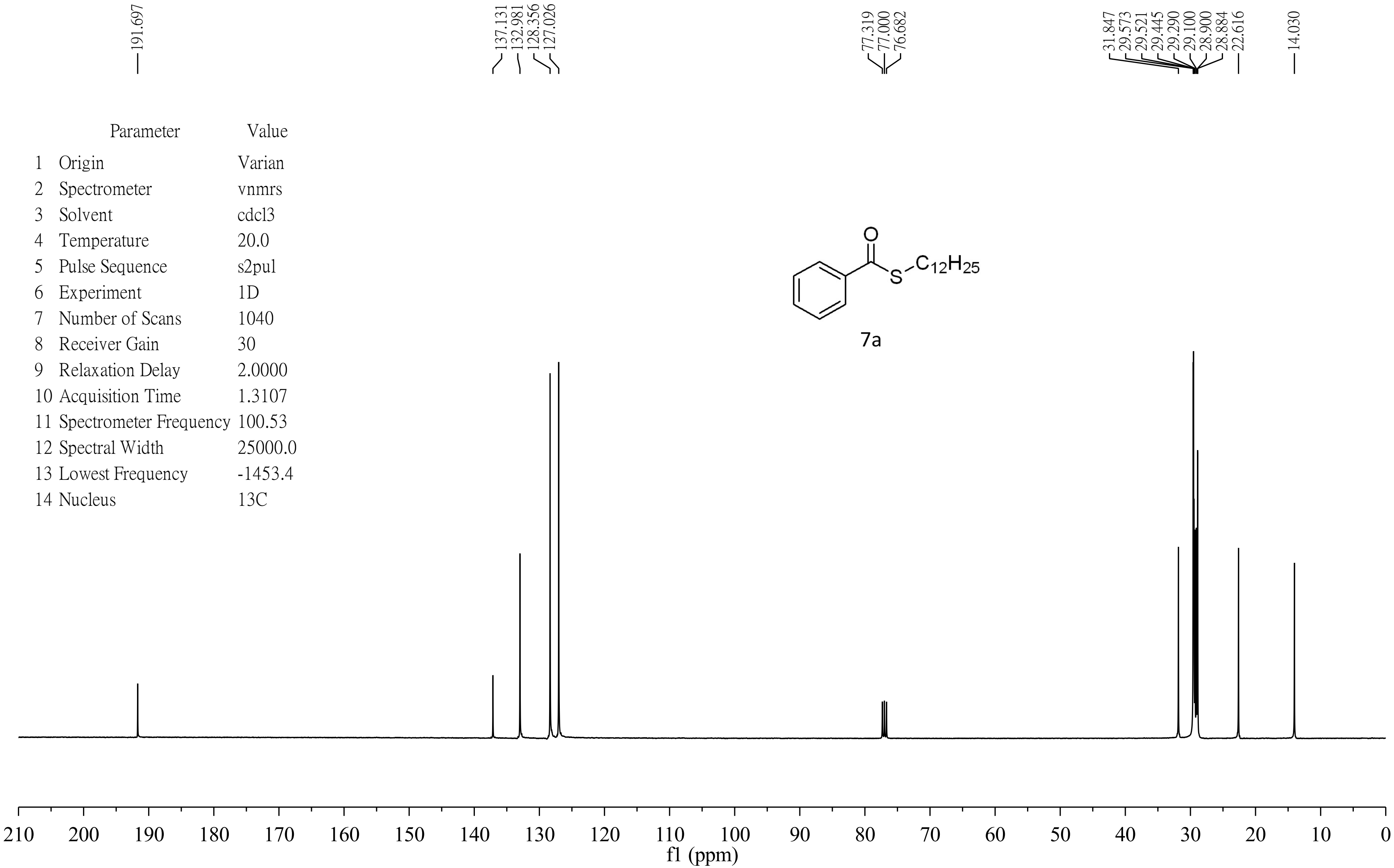

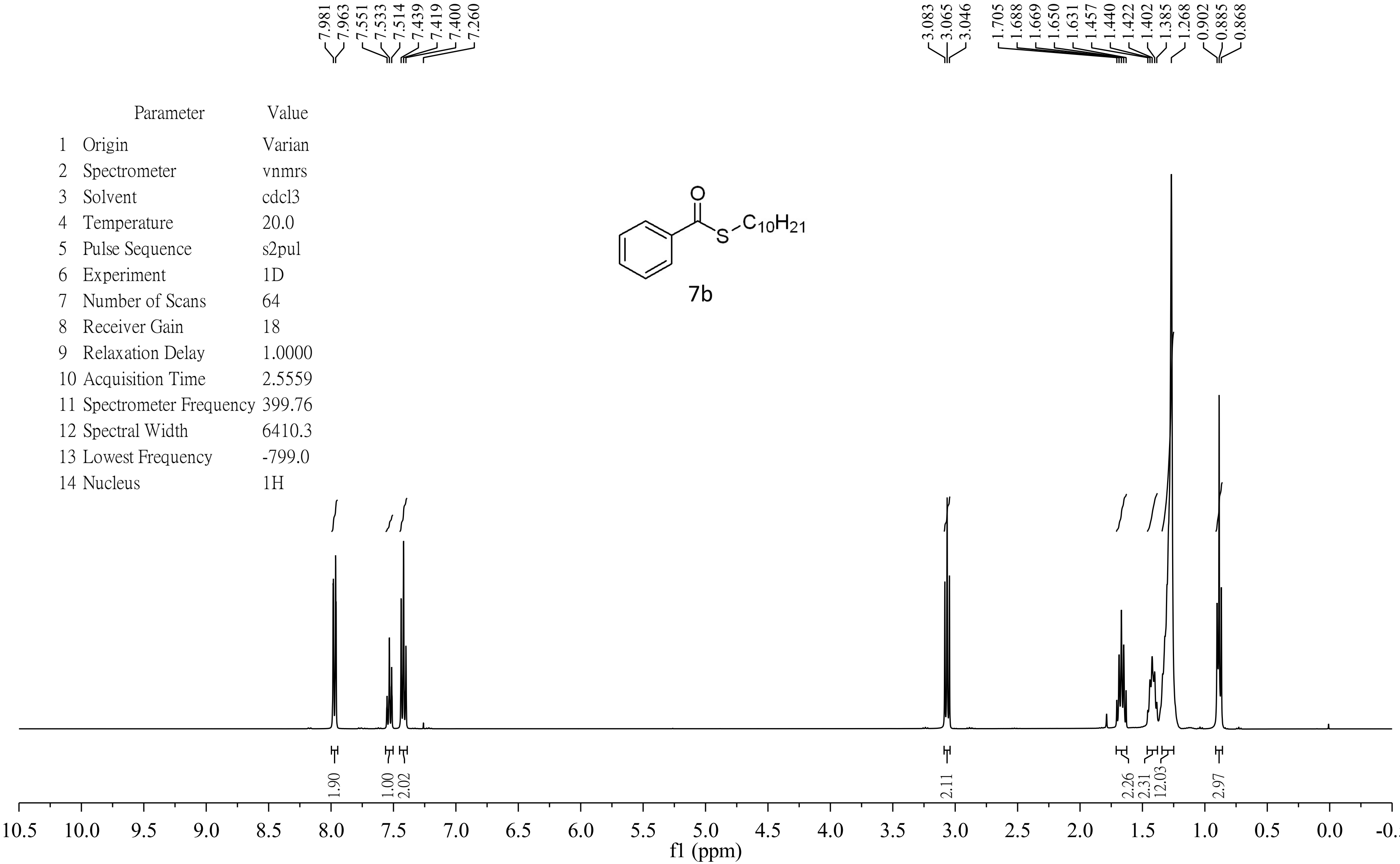

	Parameter	Value
1	Origin	Varian
2	Spectrometer	vnmrs
3	Solvent	cdcl3
4	Temperature	20.0
5	Pulse Sequence	s2pul
6	Experiment	1D
7	Number of Scans	64
8	Receiver Gain	18
9	Relaxation Delay	1.0000
10	Acquisition Time	2.5559
11	Spectrometer Frequency	399.76
12	Spectral Width	6410.3
13	Lowest Frequency	-799.0
14	Nucleus	1H

	Parameter	Value
1	Origin	Varian
2	Spectrometer	vnmrs
3	Solvent	cdcl3
4	Temperature	20.0
5	Pulse Sequence	s2pul
6	Experiment	1D
7	Number of Scans	684
8	Receiver Gain	30
9	Relaxation Delay	2.0000
10	Acquisition Time	1.3107
11	Spectrometer Frequency	100.53
12	Spectral Width	25000.0
13	Lowest Frequency	-1450.3
14	Nucleus	13C

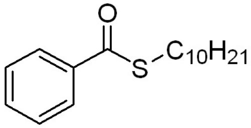

7b

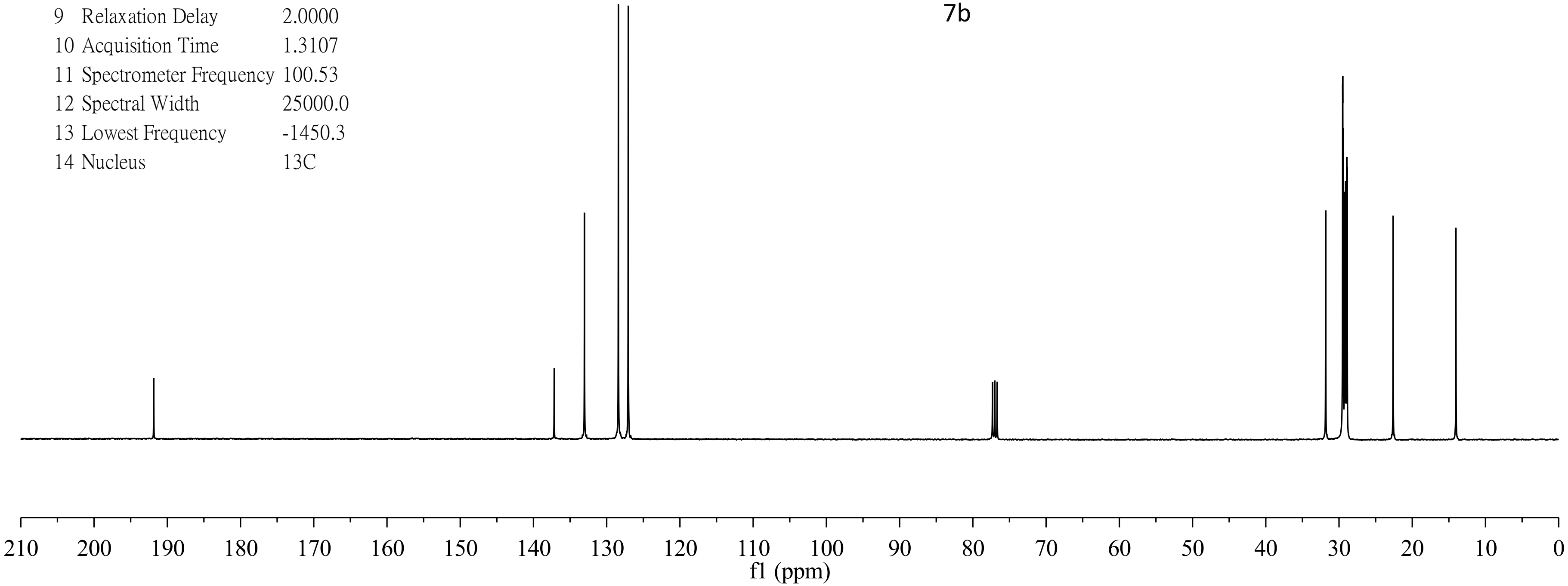

Parameter	Value
Origin	Varian
Spectrometer	vnmrs
Solvent	cdcl3
Temperature	20.0
Pulse Sequence	s2pul
Experiment	1D
Number of Scans	52
Receiver Gain	6
Relaxation Delay	1.0000
Acquisition Time	2.5559
Spectrometer Frequency	399.76
Spectral Width	6410.3
Lowest Frequency	-806.6
Nucleus	1H

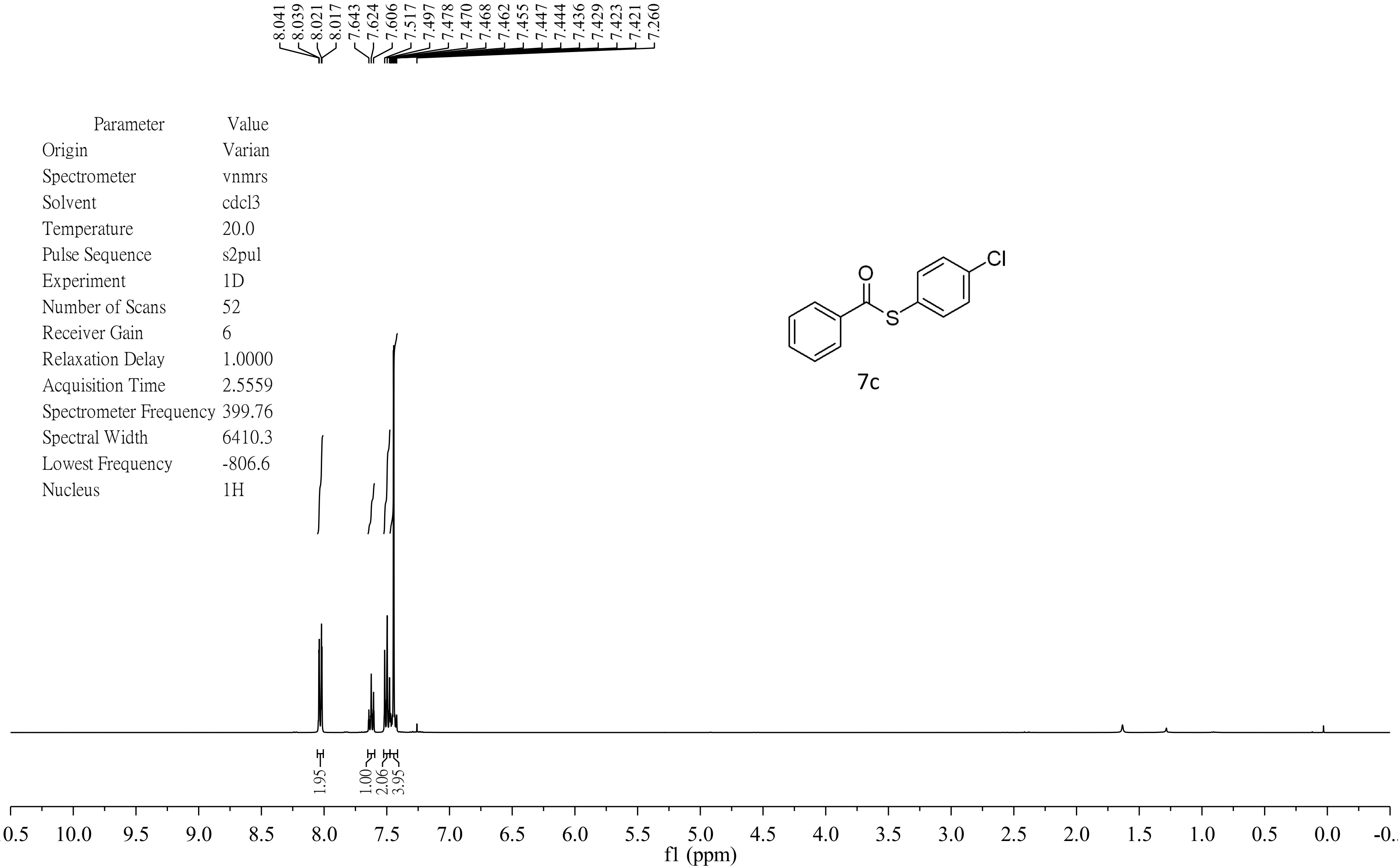

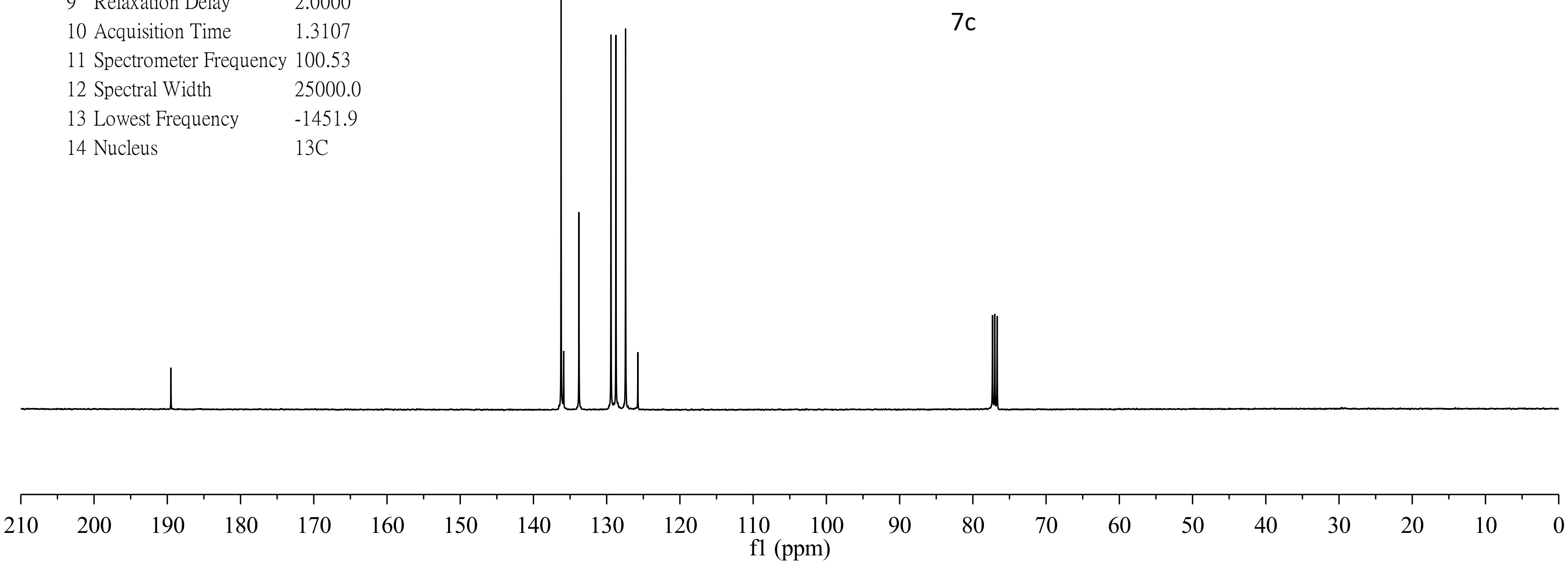

	Parameter	Value
1	Origin	Varian
2	Spectrometer	vnmrs
3	Solvent	cdcl3
4	Temperature	20.0
5	Pulse Sequence	s2pul
6	Experiment	1D
7	Number of Scans	1040
8	Receiver Gain	30
9	Relaxation Delay	2.0000
10	Acquisition Time	1.3107
11	Spectrometer Frequency	100.53
12	Spectral Width	25000.0
13	Lowest Frequency	-1451.9
14	Nucleus	13C

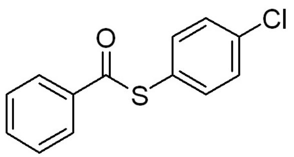

7c

	Parameter	Value
1	Origin	Varian
2	Spectrometer	vnmrs
3	Solvent	cdcl3
4	Temperature	20.0
5	Pulse Sequence	s2pul
6	Experiment	1D
7	Number of Scans	128
8	Receiver Gain	30
9	Relaxation Delay	1.0000
10	Acquisition Time	2.5559
11	Spectrometer Frequency	399.76
12	Spectral Width	6410.3
13	Lowest Frequency	-800.2
14	Nucleus	1H

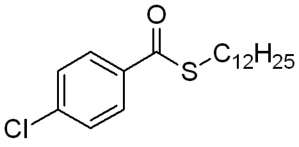

7d

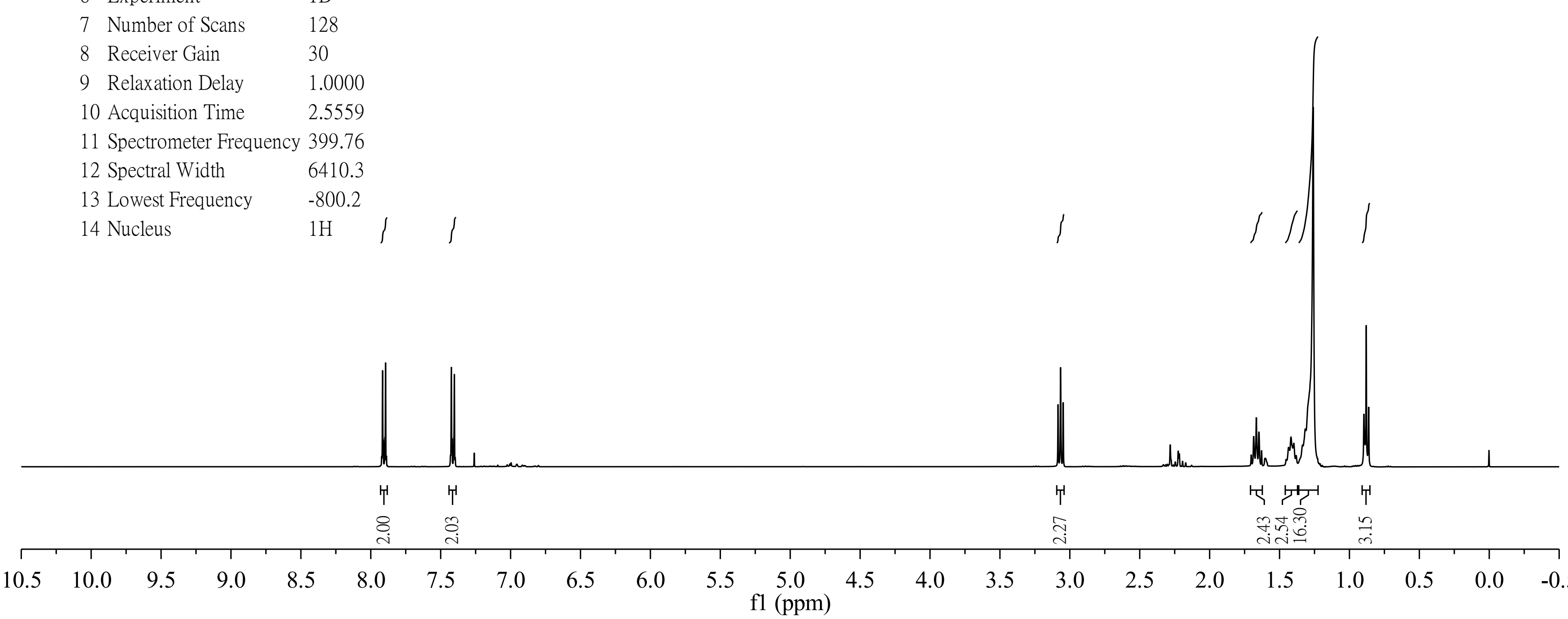

	Parameter	Value
1	Origin	Varian
2	Spectrometer	vnmrs
3	Solvent	cdcl3
4	Temperature	20.0
5	Pulse Sequence	s2pul
6	Experiment	1D
7	Number of Scans	1360
8	Receiver Gain	30
9	Relaxation Delay	2.0000
10	Acquisition Time	1.3107
11	Spectrometer Frequency	100.53
12	Spectral Width	25000.0
13	Lowest Frequency	-1443.1
14	Nucleus	13C

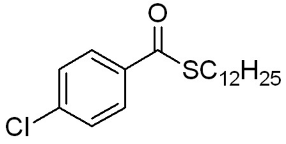

7d

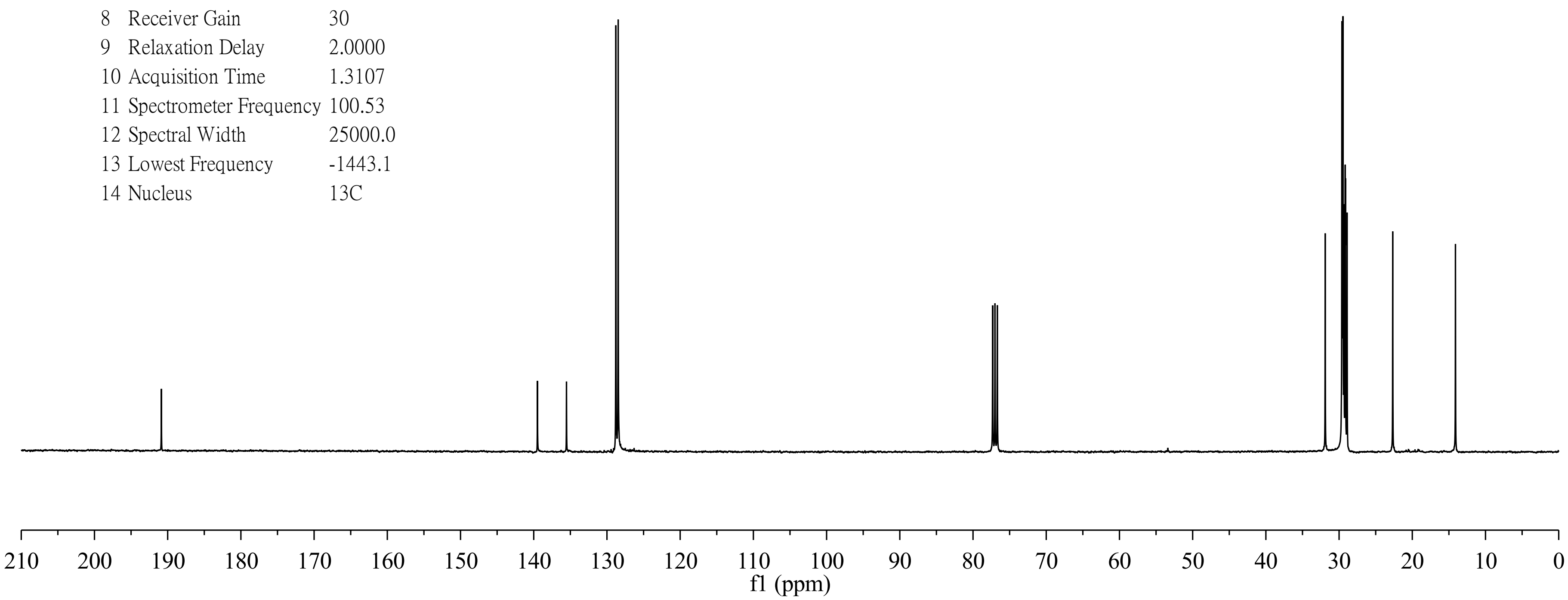

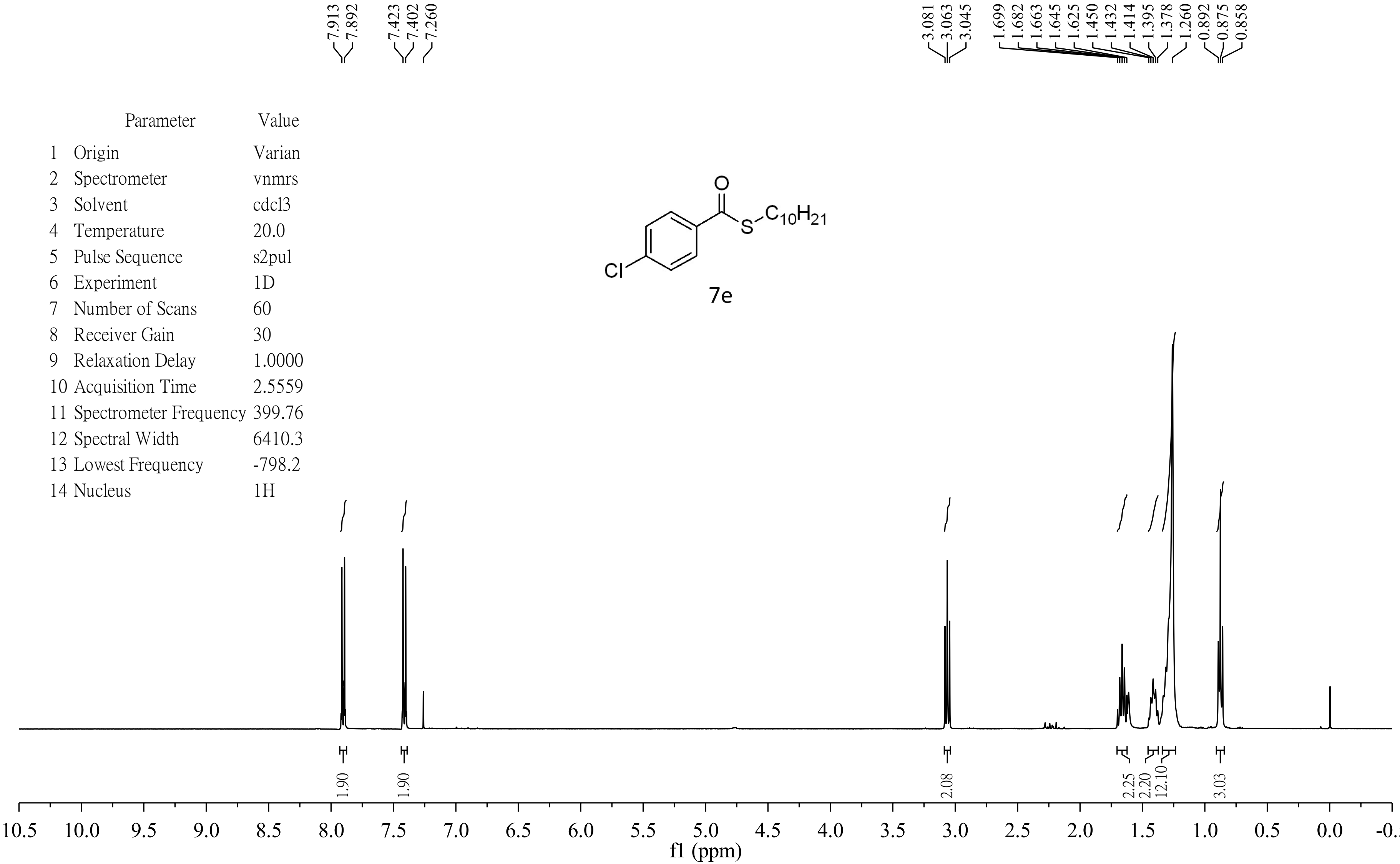
7.913
7.892
7.423
7.402
7.260
3.081
3.063
3.045
1.699
1.682
1.663
1.645
1.625
1.450
1.432
1.414
1.395
1.378
1.260
0.892
0.875
0.858
Parameter Value
1 Origin Varian
2 Spectrometer vnmrs
3 Solvent cdcl3
4 Temperature 20.0
5 Pulse Sequence s2pul
6 Experiment 1D
7 Number of Scans 60
8 Receiver Gain 30
9 Relaxation Delay 1.0000
10 Acquisition Time 2.5559
11 Spectrometer Frequency 399.76
12 Spectral Width 6410.3
13 Lowest Frequency -798.2
14 Nucleus 1H
O
S
C10H21
Cl
7e
1.90
1.90
2.08
2.25
2.20
12.10
3.03
10.5 10.0 9.5 9.0 8.5 8.0 7.5 7.0 6.5 6.0 5.5 5.0 4.5 4.0 3.5 3.0 2.5 2.0 1.5 1.0 0.5 0.0 -0.5
f1 (ppm)

	Parameter	Value
1	Origin	Varian
2	Spectrometer	vnmrs
3	Solvent	cdcl3
4	Temperature	20.0
5	Pulse Sequence	s2pul
6	Experiment	1D
7	Number of Scans	1320
8	Receiver Gain	30
9	Relaxation Delay	2.0000
10	Acquisition Time	1.3107
11	Spectrometer Frequency	100.53
12	Spectral Width	25000.0
13	Lowest Frequency	-1441.8
14	Nucleus	13C

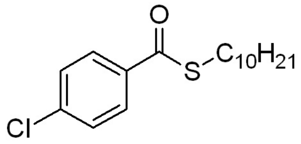

7e

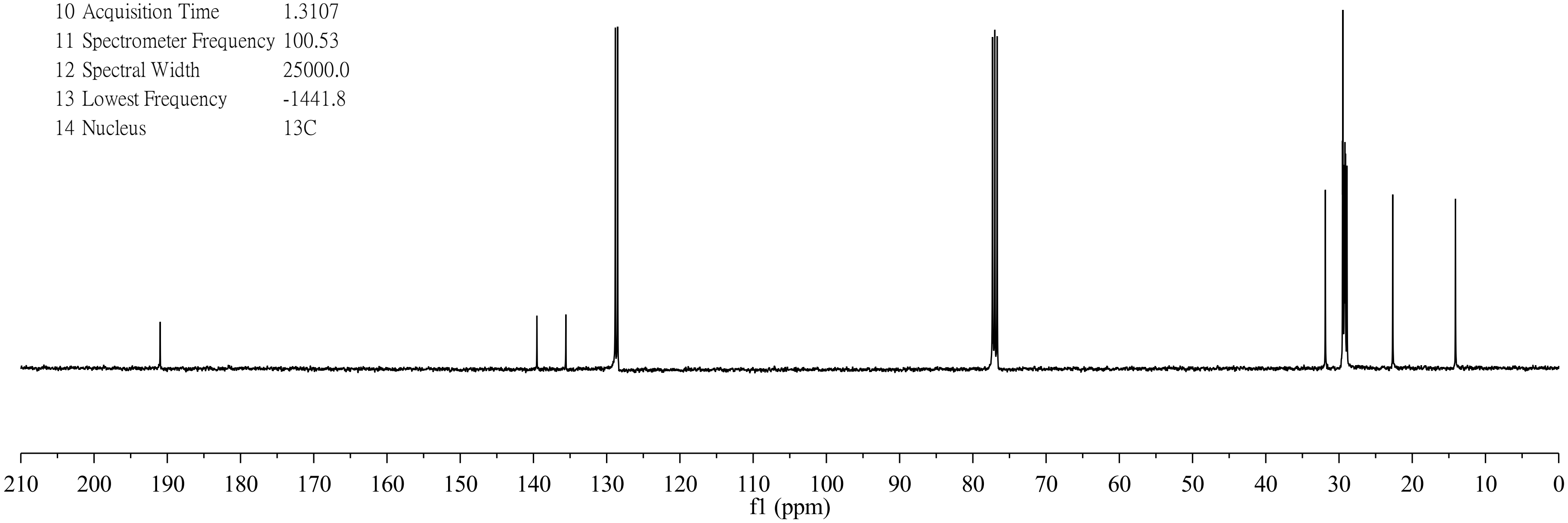

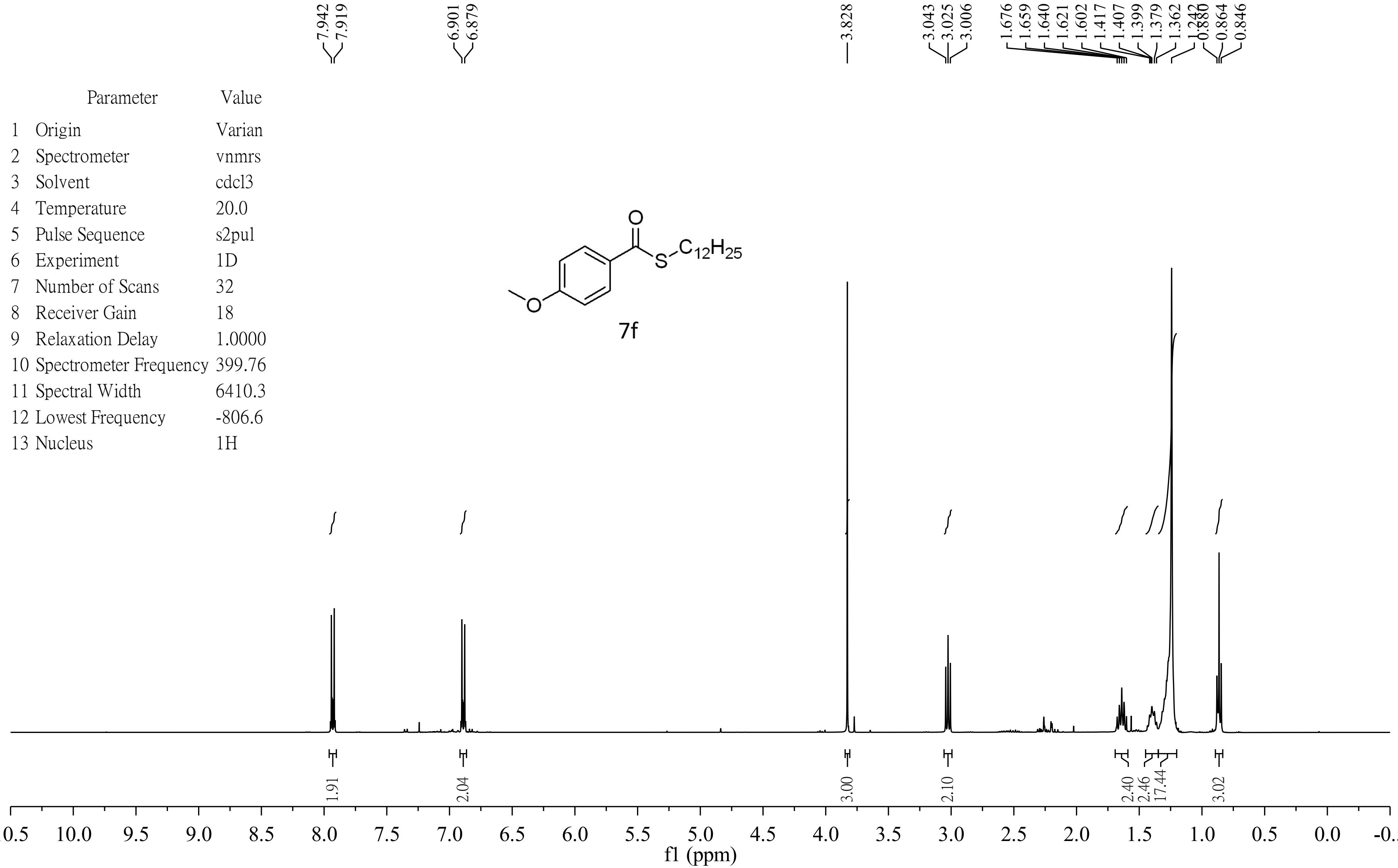

	Parameter	Value
1	Origin	Varian
2	Spectrometer	vnmrs
3	Solvent	cdcl3
4	Temperature	20.0
5	Pulse Sequence	s2pul
6	Experiment	1D
7	Number of Scans	32
8	Receiver Gain	18
9	Relaxation Delay	1.0000
10	Spectrometer Frequency	399.76
11	Spectral Width	6410.3
12	Lowest Frequency	-806.6
13	Nucleus	1H

	Parameter	Value
1	Origin	Varian
2	Spectrometer	vnmrs
3	Solvent	cdcl3
4	Temperature	20.0
5	Pulse Sequence	s2pul
6	Experiment	1D
7	Number of Scans	104
8	Receiver Gain	30
9	Relaxation Delay	2.0000
10	Spectrometer Frequency	100.53
11	Spectral Width	25000.0
12	Lowest Frequency	-1443.1
13	Nucleus	13C

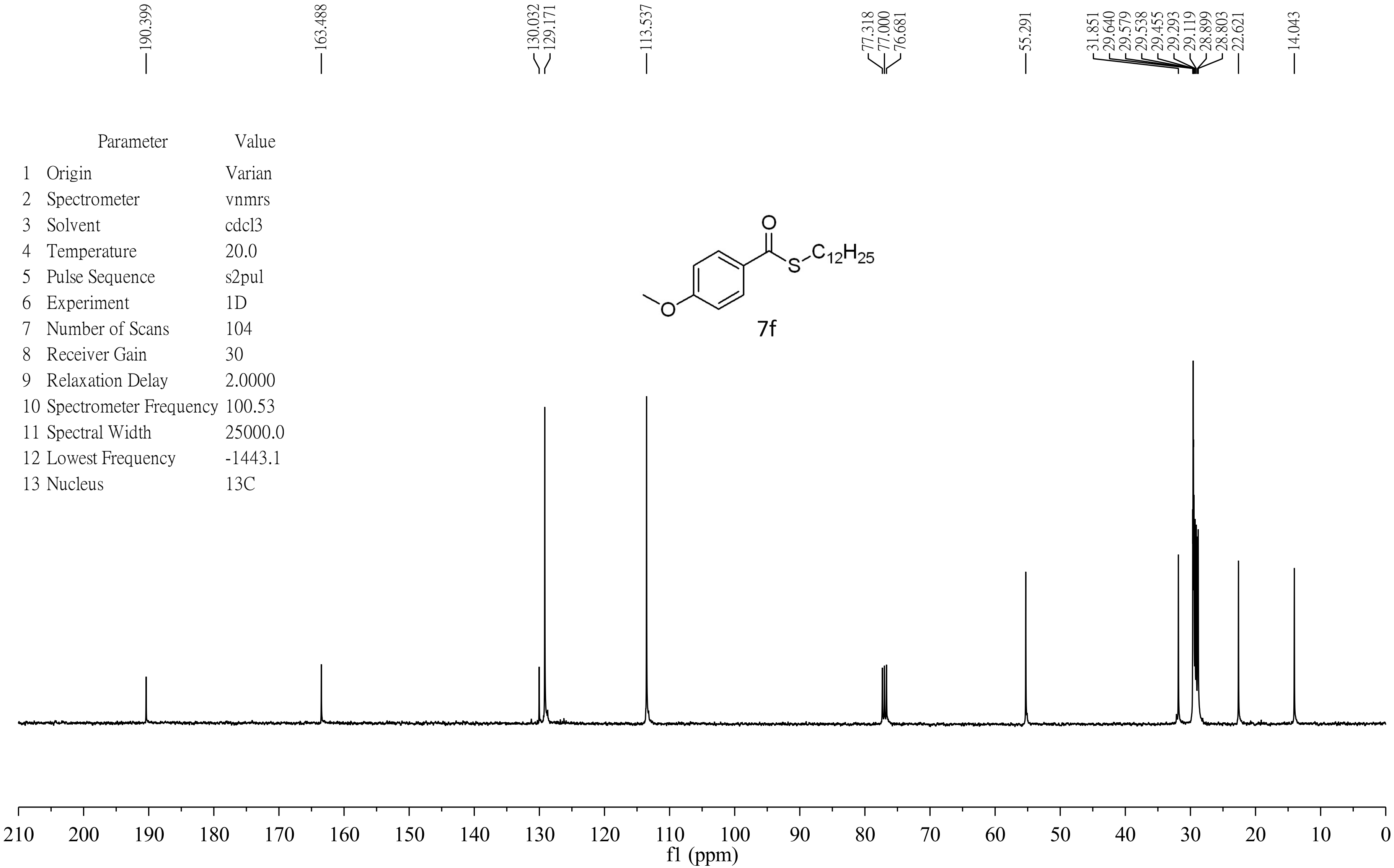

	Parameter	Value
1	Origin	Varian
2	Spectrometer	vnmrs
3	Solvent	cdcl3
4	Temperature	20.0
5	Pulse Sequence	s2pul
6	Experiment	1D
7	Number of Scans	40
8	Receiver Gain	30
9	Relaxation Delay	1.0000
10	Spectrometer Frequency	399.76
11	Spectral Width	6410.3
12	Lowest Frequency	-806.6
13	Nucleus	1H

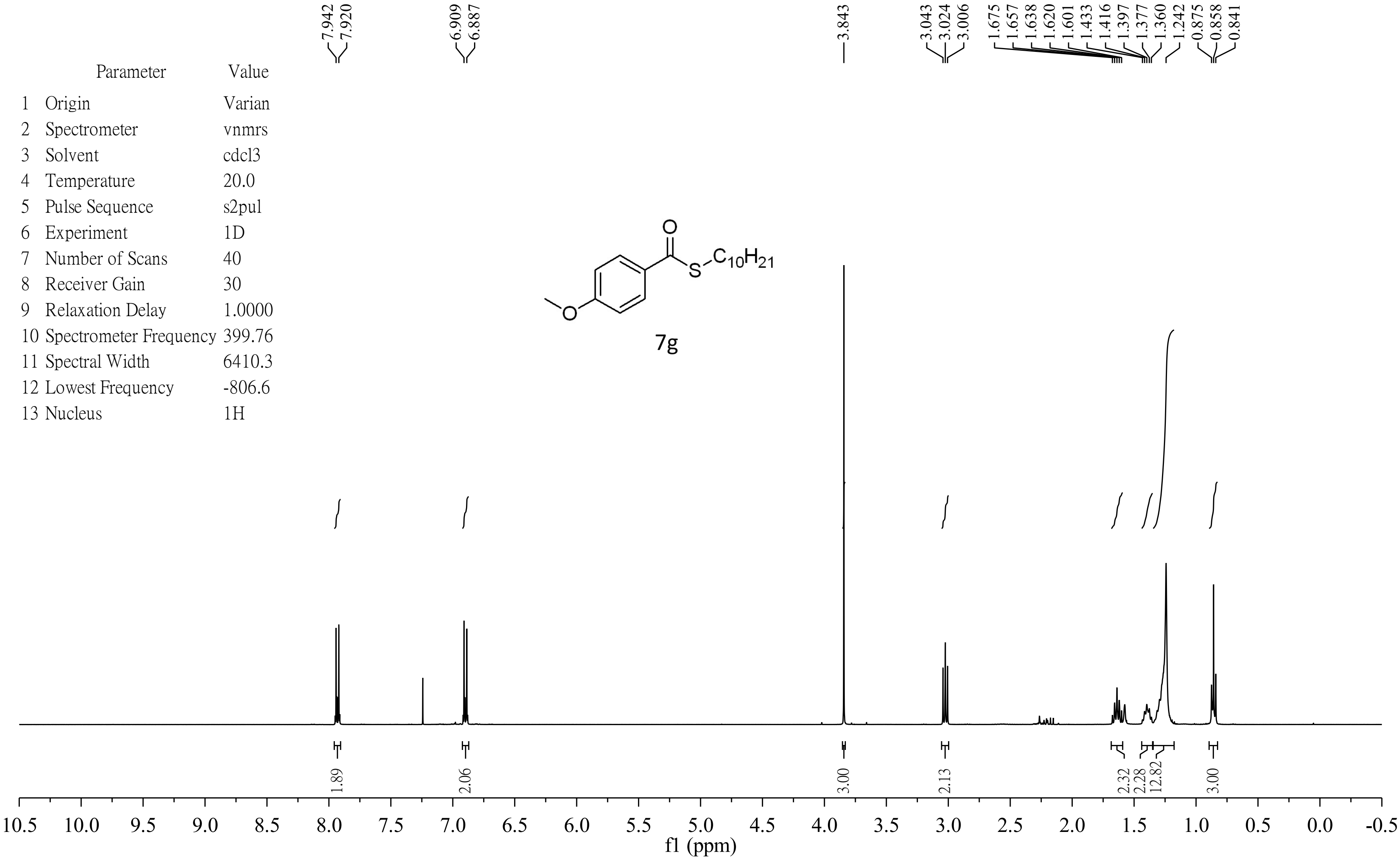

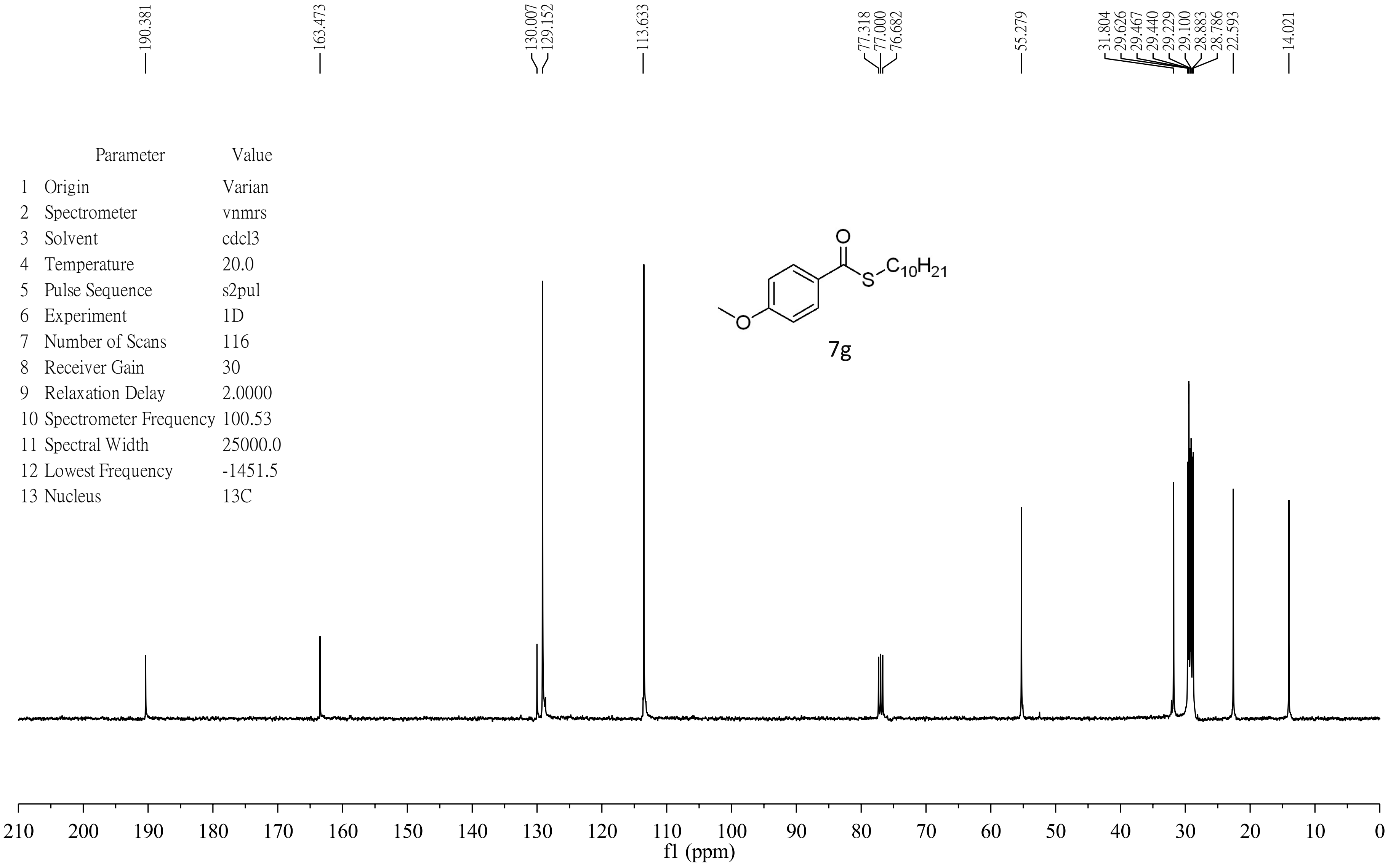

	Parameter	Value
1	Origin	Varian
2	Spectrometer	vnmrs
3	Solvent	cdcl3
4	Temperature	20.0
5	Pulse Sequence	s2pul
6	Experiment	1D
7	Number of Scans	116
8	Receiver Gain	30
9	Relaxation Delay	2.0000
10	Spectrometer Frequency	100.53
11	Spectral Width	25000.0
12	Lowest Frequency	-1451.5
13	Nucleus	13C

7g

	Parameter	Value
1	Origin	Varian
2	Spectrometer	vnmrs
3	Solvent	cdcl3
4	Temperature	20.0
5	Pulse Sequence	s2pul
6	Experiment	1D
7	Number of Scans	44
8	Receiver Gain	18
9	Relaxation Delay	1.0000
10	Spectrometer Frequency	399.76
11	Spectral Width	6410.3
12	Lowest Frequency	-806.6
13	Nucleus	1H

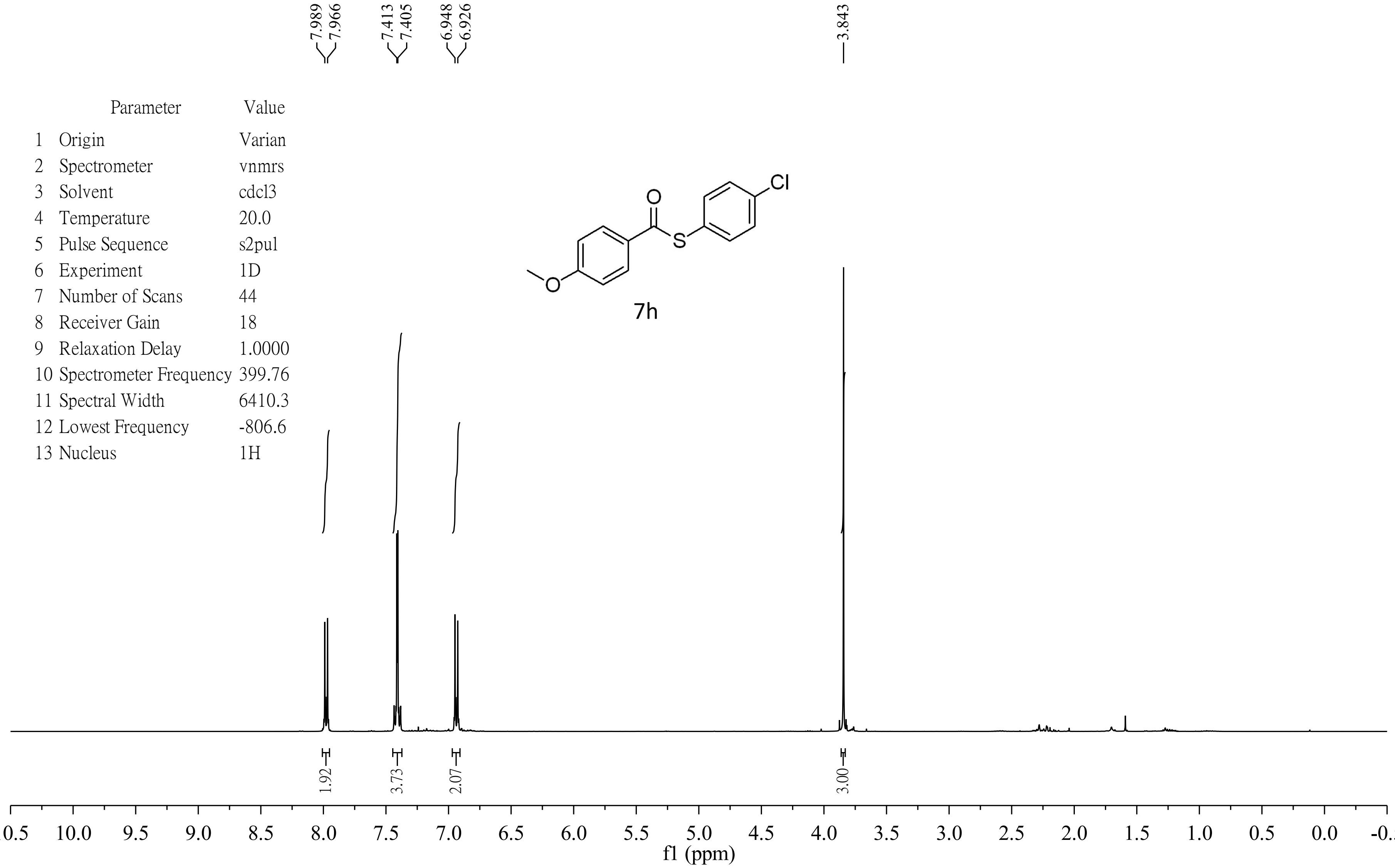

187.735
163.967
136.232
135.566
129.583
129.216
128.863
126.046
113.825
77.319
77.000
76.682
55.380

	Parameter	Value
1	Origin	Varian
2	Spectrometer	vnmrs
3	Solvent	cdcl3
4	Temperature	20.0
5	Pulse Sequence	s2pul
6	Experiment	1D
7	Number of Scans	280
8	Receiver Gain	30
9	Relaxation Delay	2.0000
10	Spectrometer Frequency	100.53
11	Spectral Width	25000.0
12	Lowest Frequency	-1459.1
13	Nucleus	13C

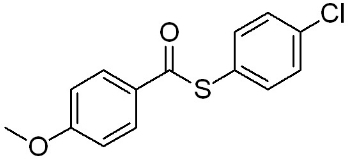

7h

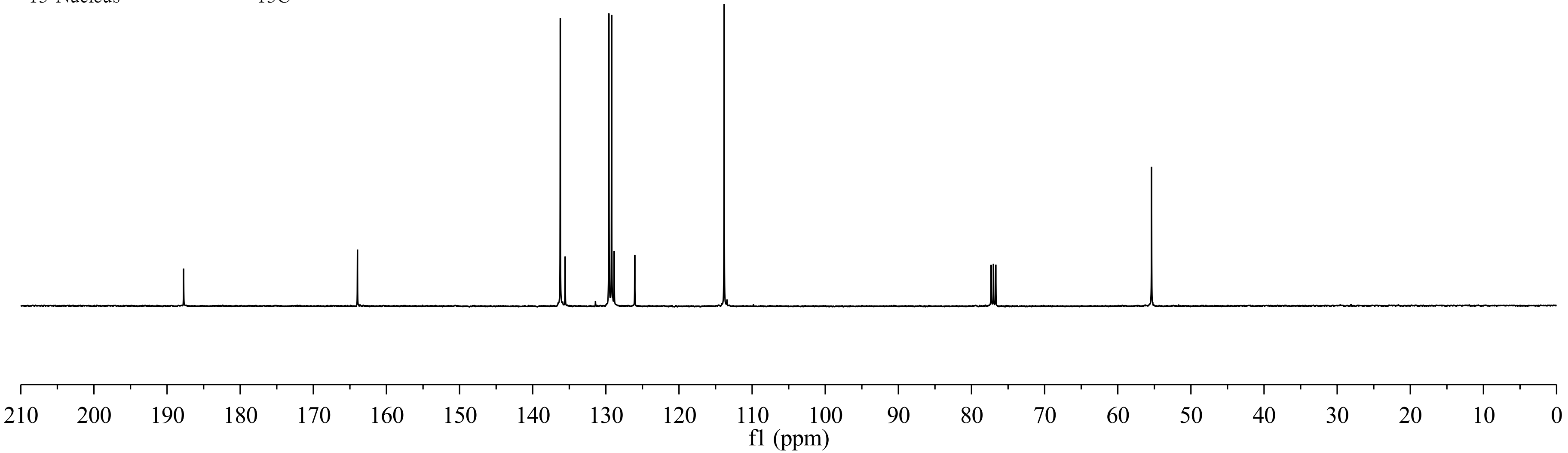

	Parameter	Value
1	Origin	Varian
2	Spectrometer	vnmrs
3	Solvent	cdcl3
4	Temperature	20.0
5	Pulse Sequence	s2pul
6	Experiment	1D
7	Number of Scans	88
8	Receiver Gain	18
9	Relaxation Delay	1.0000
10	Acquisition Time	2.5559
11	Spectrometer Frequency	399.76
12	Spectral Width	6410.3
13	Lowest Frequency	-799.4
14	Nucleus	1H

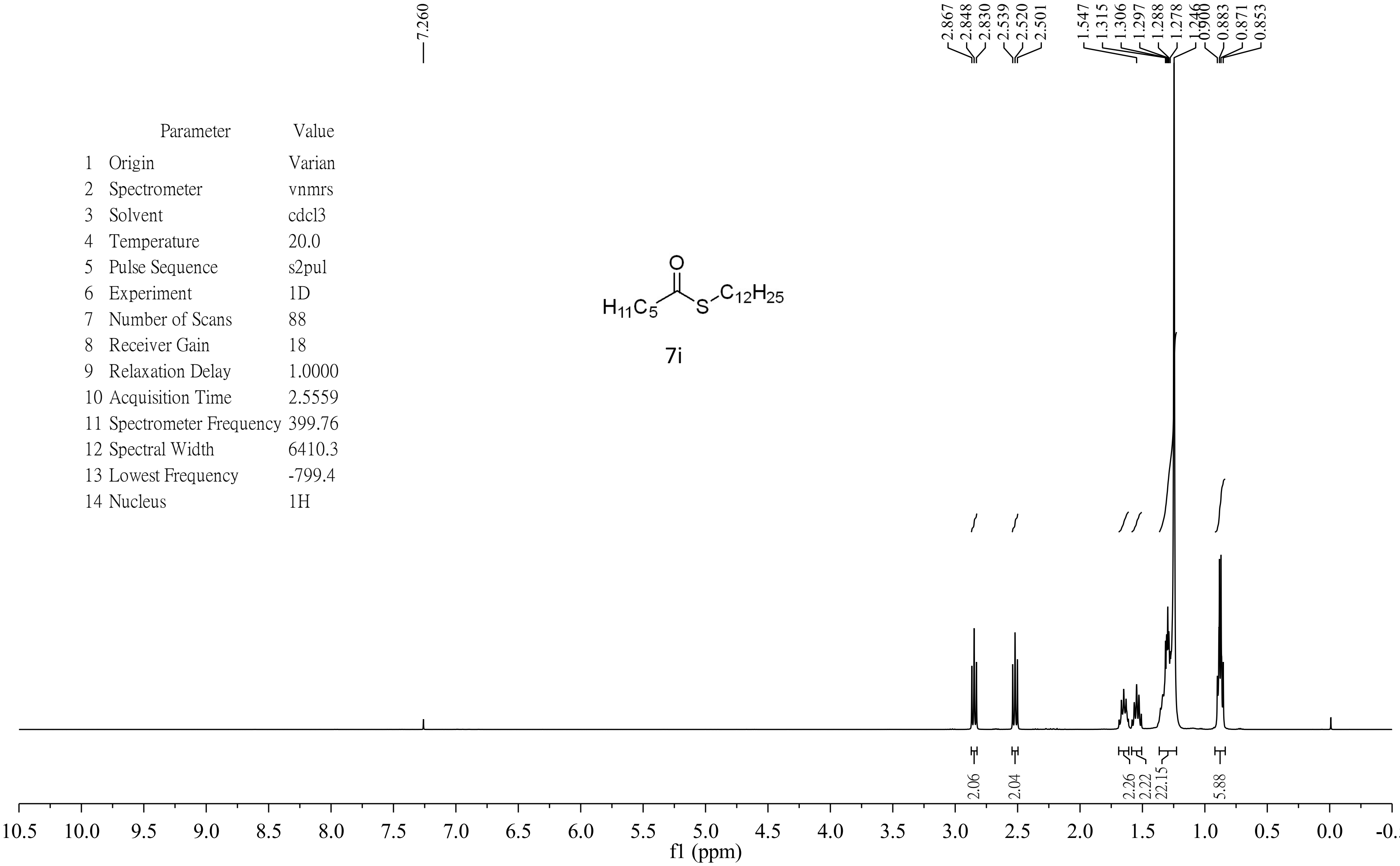

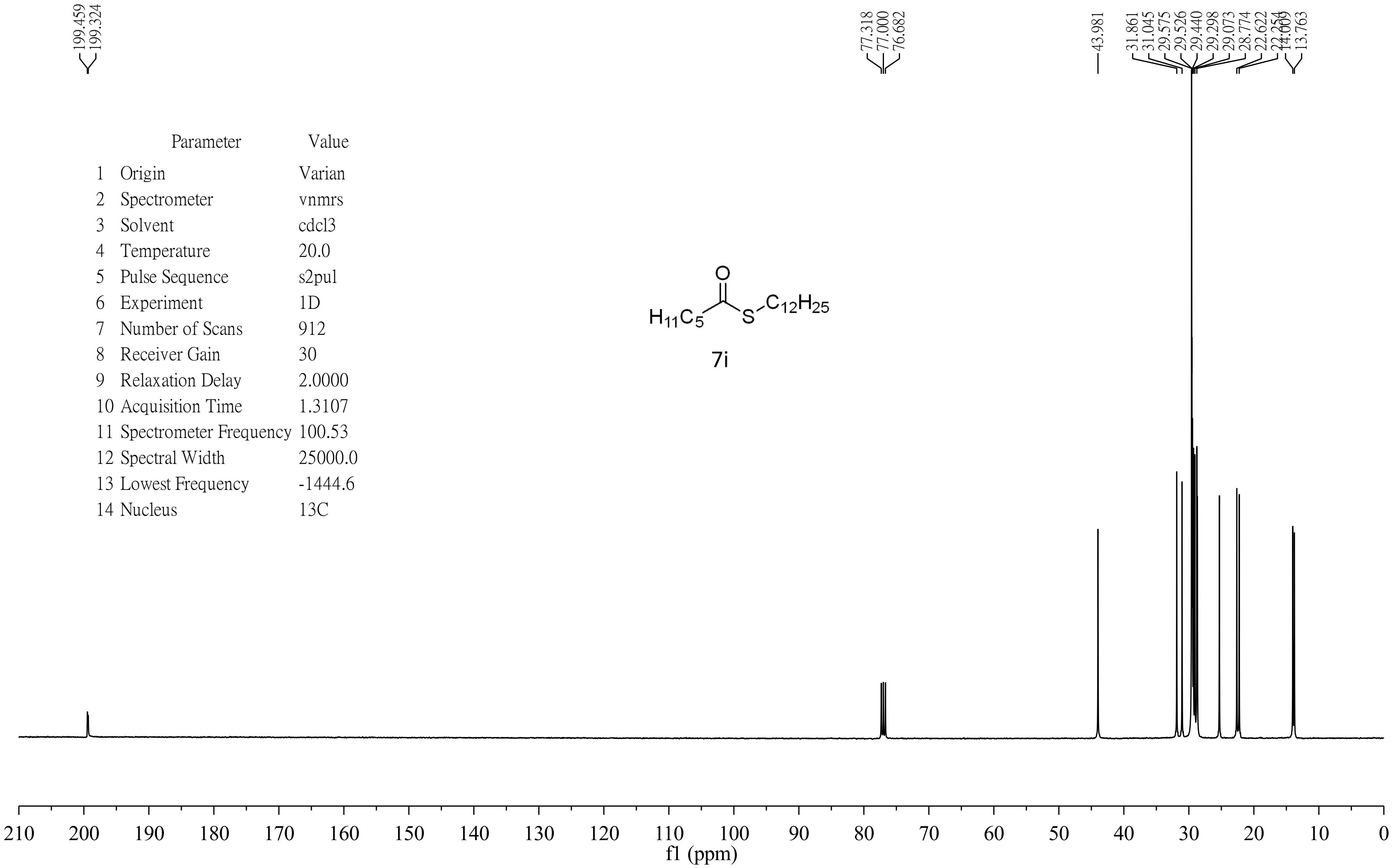
199.459
199.324
77.318
77.000
76.682
43.981
31.861
31.045
29.575
29.526
29.440
29.298
29.073
28.774
22.622
22.254
14.009
13.763
Parameter Value
1 Origin Varian
2 Spectrometer vnmrs
3 Solvent cdcl3
4 Temperature 20.0
5 Pulse Sequence s2pul
6 Experiment 1D
7 Number of Scans 912
8 Receiver Gain 30
9 Relaxation Delay 2.0000
10 Acquisition Time 1.3107
11 Spectrometer Frequency 100.53
12 Spectral Width 25000.0
13 Lowest Frequency -1444.6
14 Nucleus 13C
O
H11C5
S
C12H25
7i
210 200 190 180 170 160 150 140 130 120 110 100 90 80 70 60 50 40 30 20 10 0
f1 (ppm)

Parameter	Value
Origin	Varian
Spectrometer	vnmrs
Solvent	cdcl3
Temperature	20.0
Pulse Sequence	s2pul
Experiment	1D
Number of Scans	56
Receiver Gain	30
Relaxation Delay	1.0000
Acquisition Time	2.5559
Spectrometer Frequency	399.76
Spectral Width	6410.3
Lowest Frequency	-806.6
Nucleus	1H

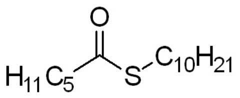

7j

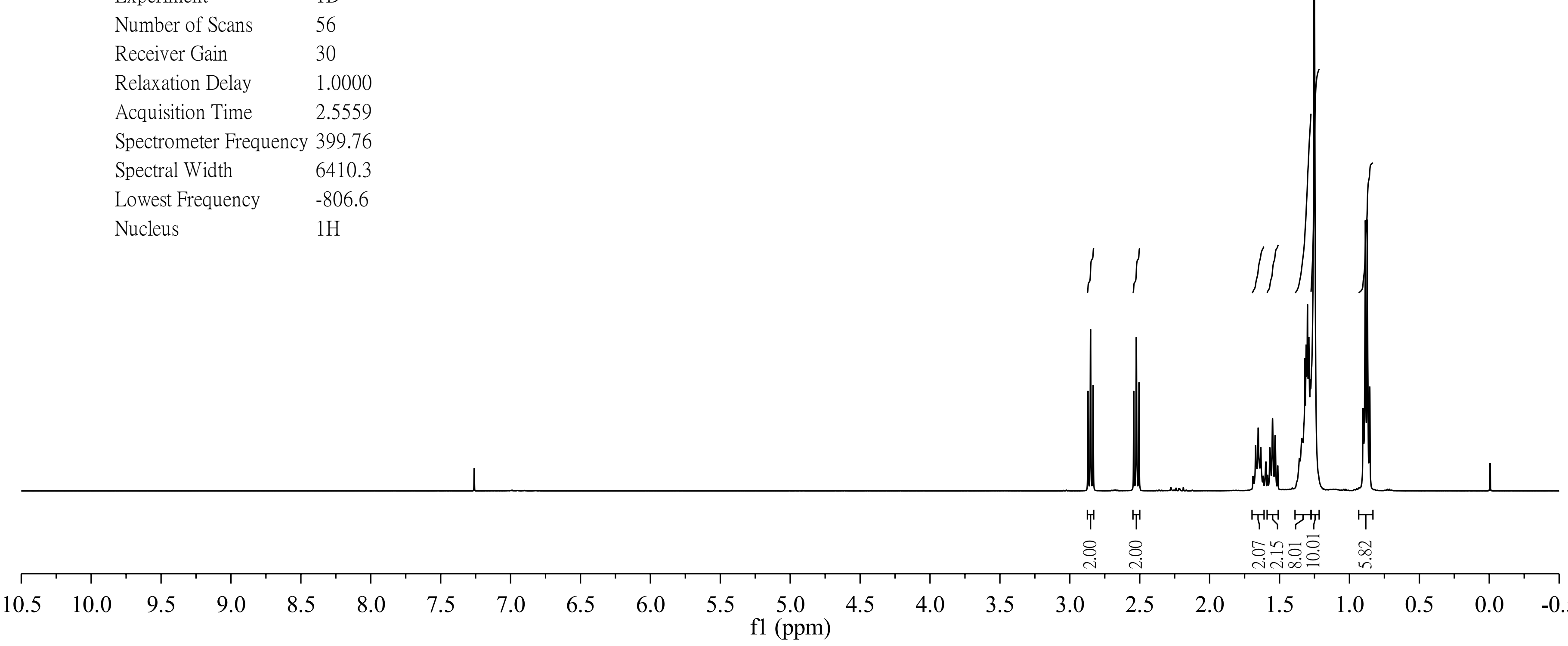

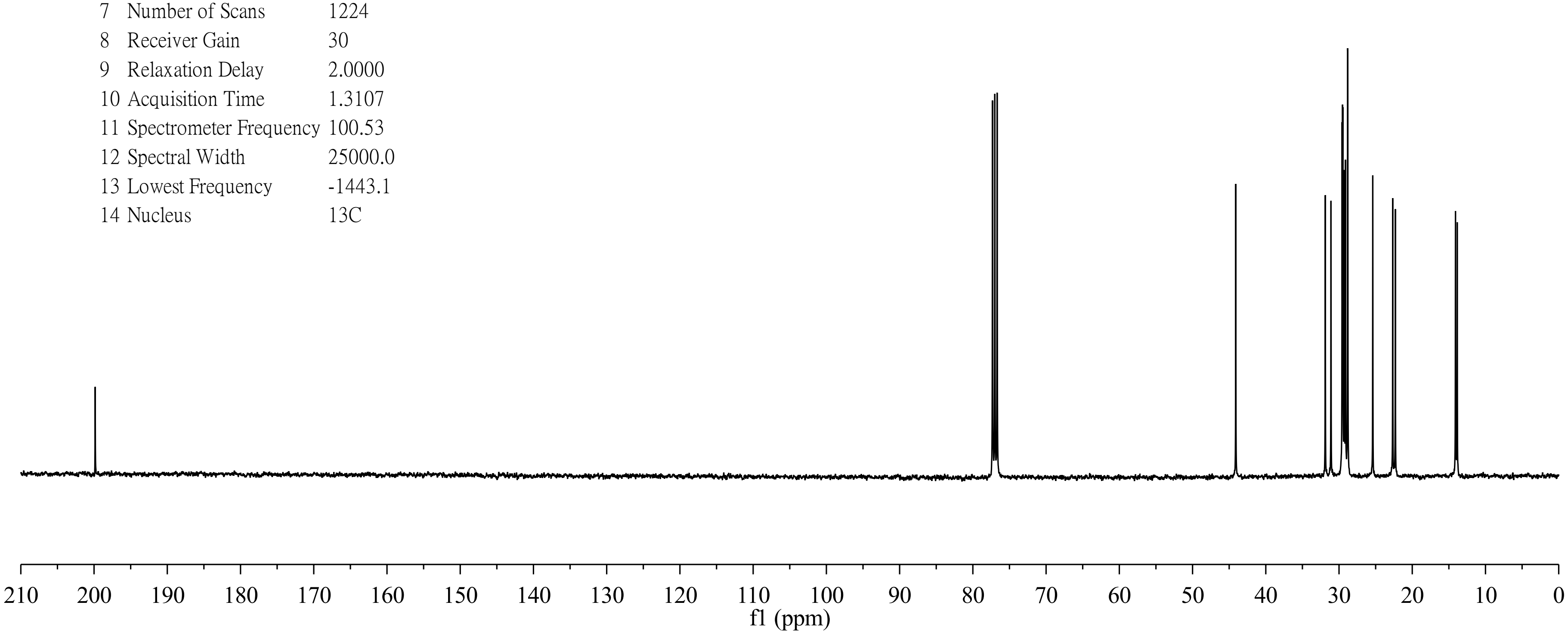

	Parameter	Value
1	Origin	Varian
2	Spectrometer	vnmrs
3	Solvent	cdcl3
4	Temperature	20.0
5	Pulse Sequence	s2pul
6	Experiment	1D
7	Number of Scans	1224
8	Receiver Gain	30
9	Relaxation Delay	2.0000
10	Acquisition Time	1.3107
11	Spectrometer Frequency	100.53
12	Spectral Width	25000.0
13	Lowest Frequency	-1443.1
14	Nucleus	13C

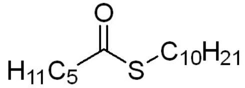

7j

	Parameter	Value
1	Origin	Varian
2	Spectrometer	vnmrs
3	Solvent	cdcl3
4	Temperature	20.0
5	Pulse Sequence	s2pul
6	Experiment	1D
7	Number of Scans	64
8	Receiver Gain	6
9	Relaxation Delay	1.0000
10	Acquisition Time	2.5559
11	Spectrometer Frequency	399.76
12	Spectral Width	6410.3
13	Lowest Frequency	-799.0
14	Nucleus	1H

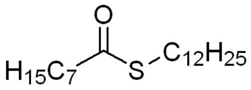

7k

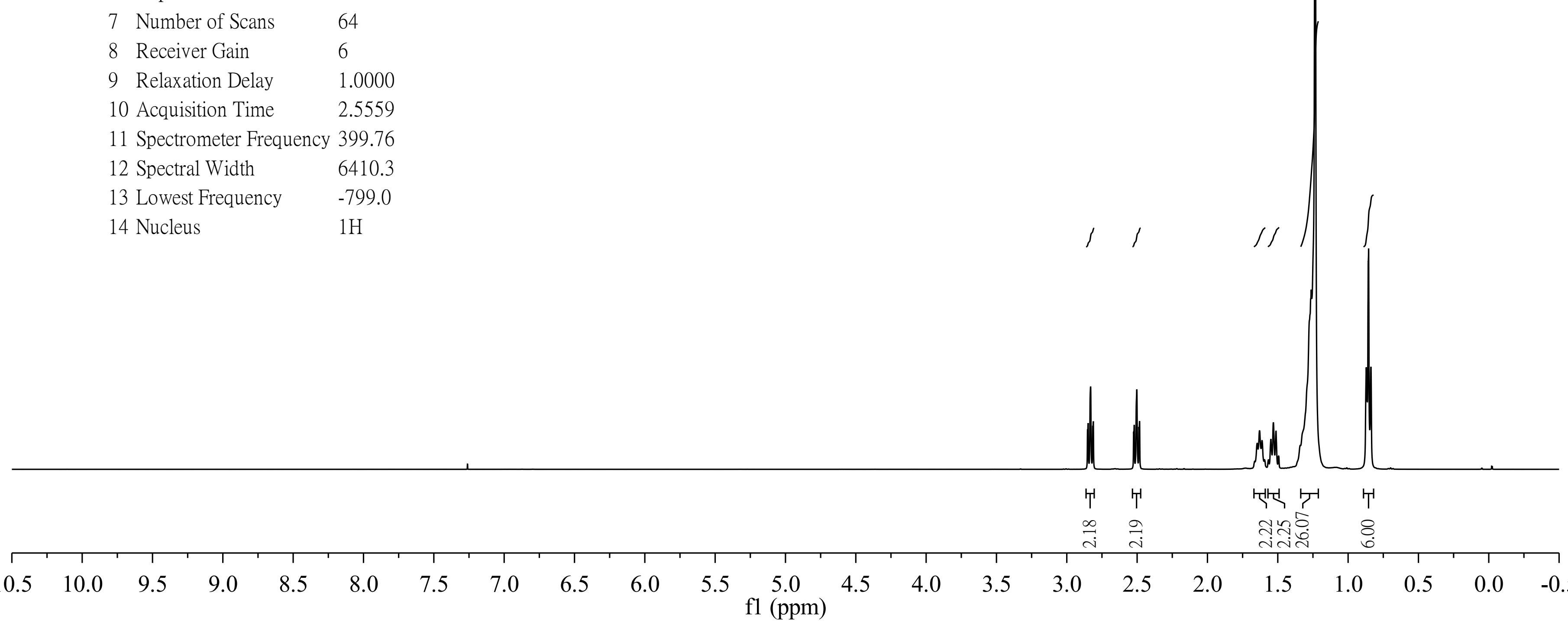

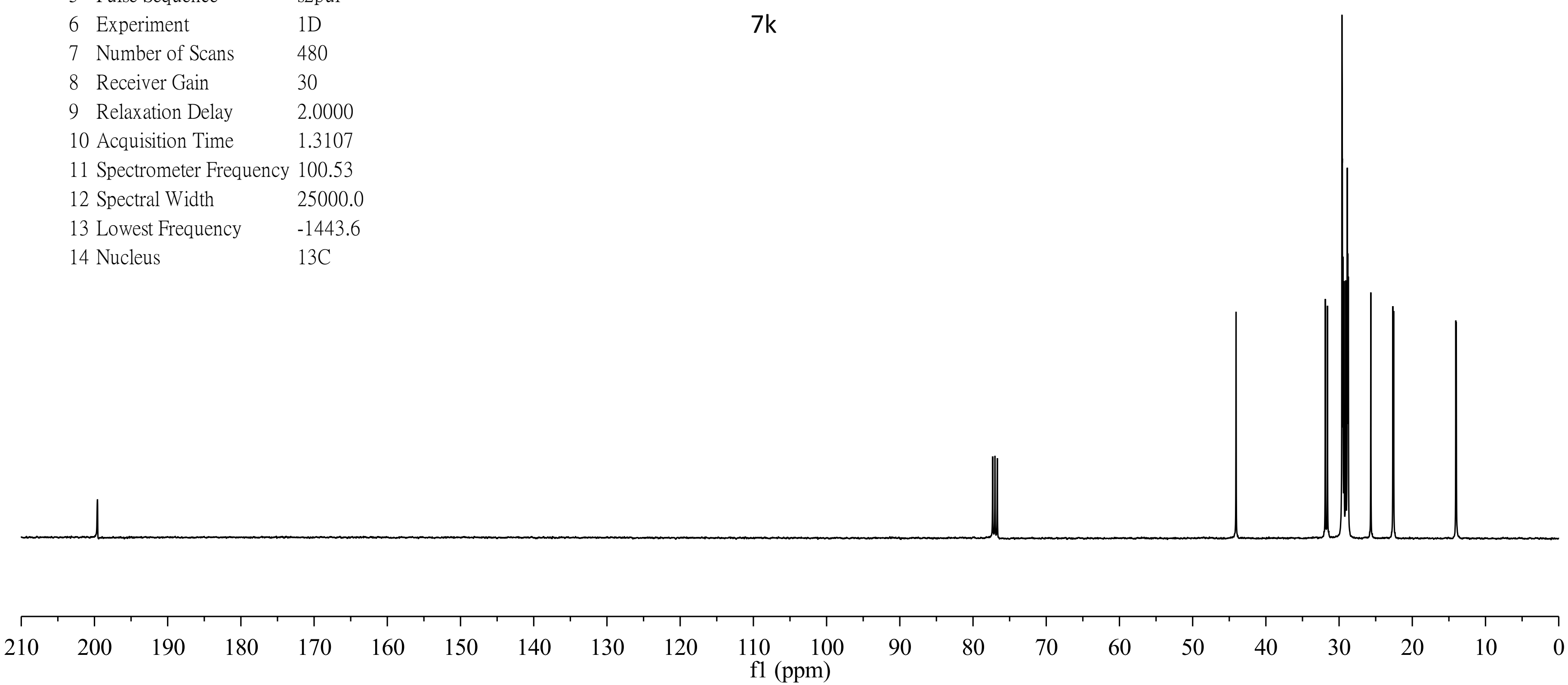

	Parameter	Value
1	Origin	Varian
2	Spectrometer	vnmrs
3	Solvent	cdcl3
4	Temperature	20.0
5	Pulse Sequence	s2pul
6	Experiment	1D
7	Number of Scans	480
8	Receiver Gain	30
9	Relaxation Delay	2.0000
10	Acquisition Time	1.3107
11	Spectrometer Frequency	100.53
12	Spectral Width	25000.0
13	Lowest Frequency	-1443.6
14	Nucleus	13C

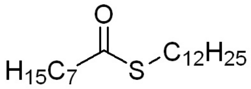

7k

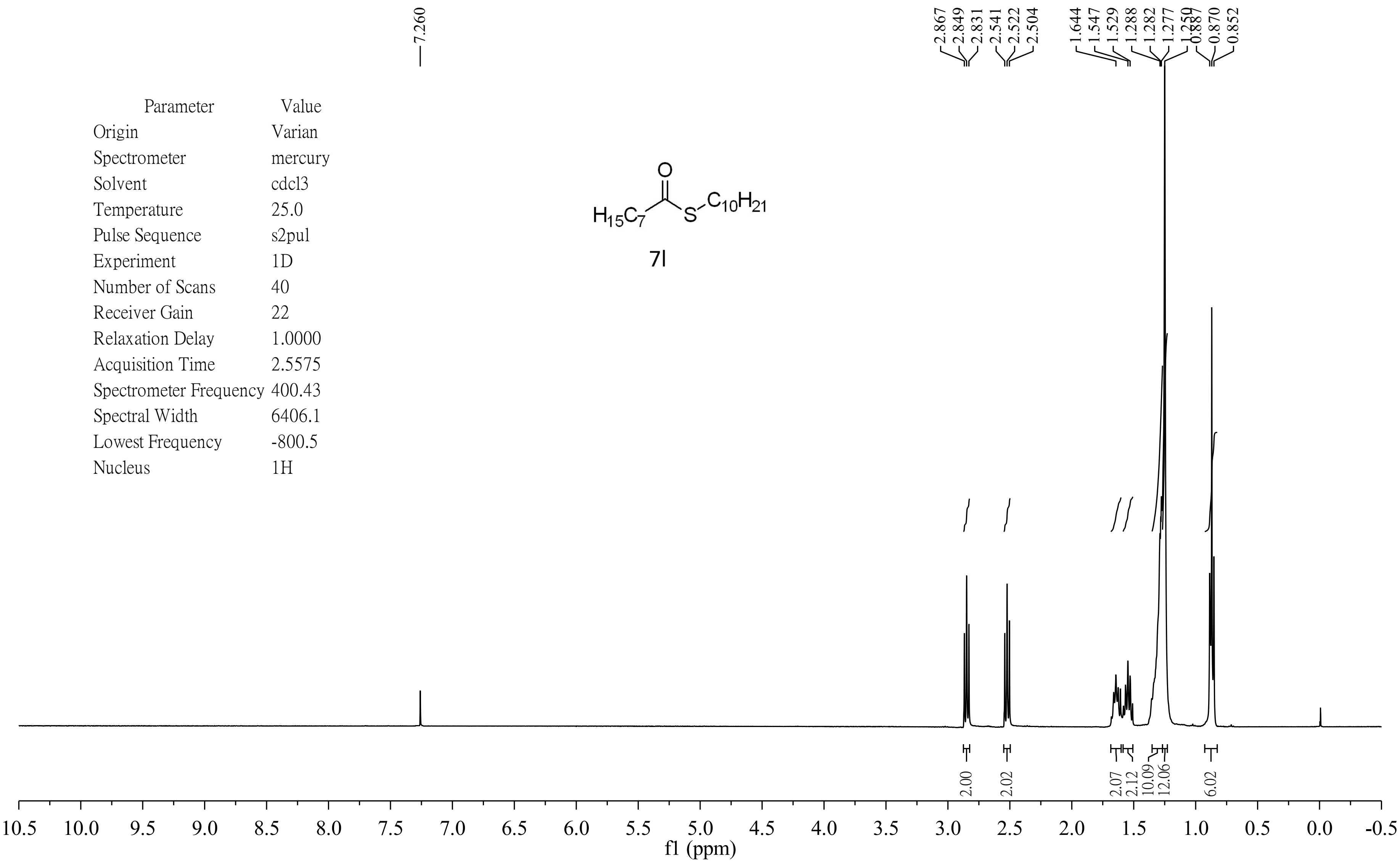
7.260
2.867
2.849
2.831
2.541
2.522
2.504
1.644
1.547
1.529
1.288
1.282
1.277
1.250
0.887
0.870
0.852
Parameter Value
Origin Varian
Spectrometer mercury
Solvent cdcl3
Temperature 25.0
Pulse Sequence s2pul
Experiment 1D
Number of Scans 40
Receiver Gain 22
Relaxation Delay 1.0000
Acquisition Time 2.5575
Spectrometer Frequency 400.43
Spectral Width 6406.1
Lowest Frequency -800.5
Nucleus 1H
O
H15C7
S
C10H21
7l
2.00
2.02
2.07
2.12
10.09
12.06
6.02
10.5 10.0 9.5 9.0 8.5 8.0 7.5 7.0 6.5 6.0 5.5 5.0 4.5 4.0 3.5 3.0 2.5 2.0 1.5 1.0 0.5 0.0 -0.5
f1 (ppm)

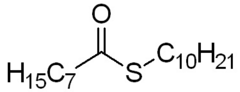

7l

	Parameter	Value
1	Origin	Varian
2	Spectrometer	vnmrs
3	Solvent	cdcl3
4	Temperature	20.0
5	Pulse Sequence	s2pul
6	Experiment	1D
7	Number of Scans	1216
8	Receiver Gain	30
9	Relaxation Delay	2.0000
10	Acquisition Time	1.3107
11	Spectrometer Frequency	100.53
12	Spectral Width	25000.0
13	Lowest Frequency	-1441.1
14	Nucleus	13C

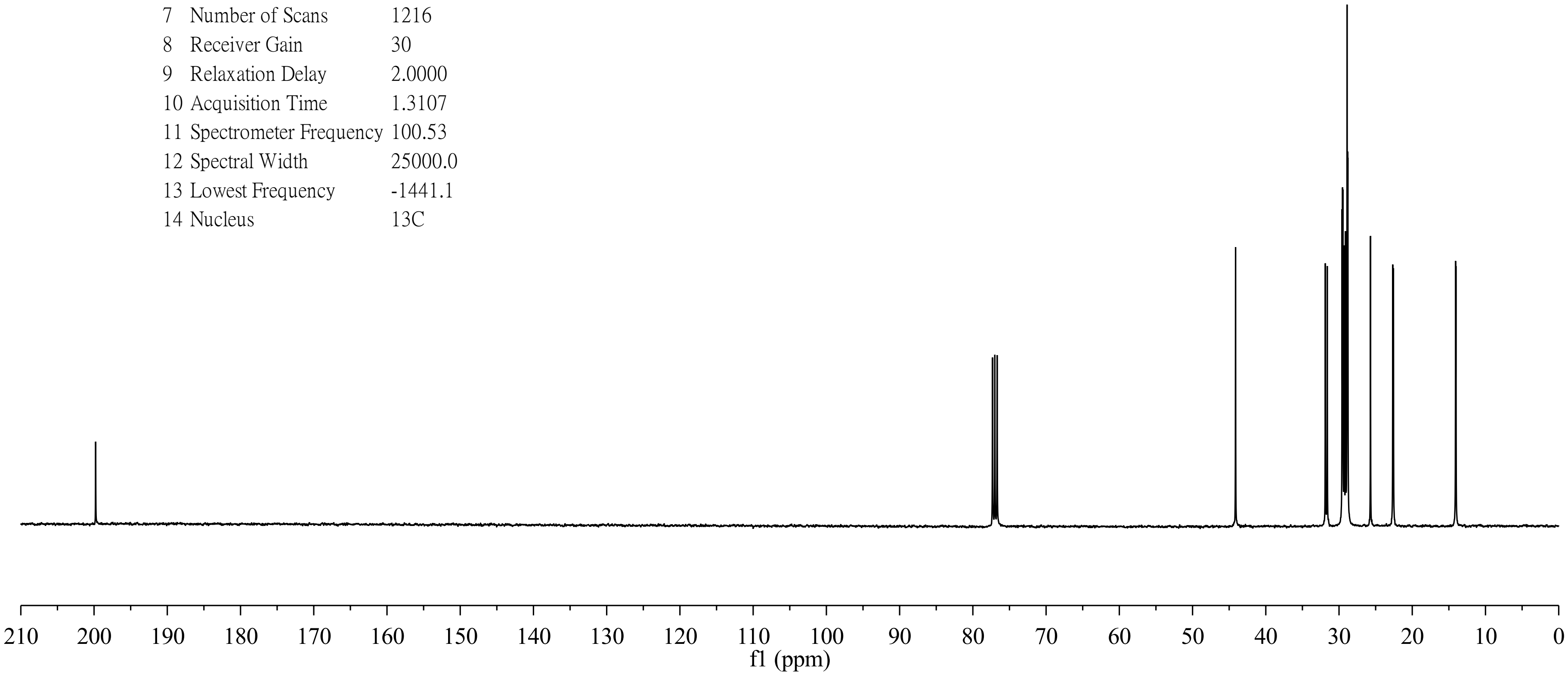

Supplement: Supplementary file 1 [file molecules-25-00352-s001.pdf]
